# GARD: Genomic Data-Based Drug Repurposing in Head and Neck Cancer with Large Language Model Validation

**DOI:** 10.3390/cancers18050757

**Published:** 2026-02-26

**Authors:** Pradham Tanikella, William Nenad, Christophe Courtine, Yifan Dai, Qingying Deng, Baiming Zou, Nosayaba Osazuwa-Peters, Travis P. Schrank, Di Wu

**Affiliations:** 1 Department of Genetics, School of Medicine, University of North Carolina at Chapel Hill, Chapel Hill, NC 27599, USA; 2 Lineberger Comprehensive Cancer Center, School of Medicine, University of North Carolina at Chapel Hill, Chapel Hill, NC 27599, USA; 3 Department of Otolaryngology/Head and Neck Surgery, School of Medicine, University of North Carolina at Chapel Hill, Chapel Hill, NC 27599, USA; 4 Department of Biostatistics, Gillings School of Global Public Health, University of North Carolina at Chapel Hill, Chapel Hill, NC 27599, USA; 5 Department of Head and Neck Surgery & Communication Sciences, Duke University School of Medicine, Durham, NC 27710, USA; 6 Department of Population Health Sciences, Duke University School of Medicine, Durham, NC 27710, USA; 7 Department of Biomedical Sciences, Adams School of Dentistry, University of North Carolina at Chapel Hill, Chapel Hill, NC 27599, USA

**Keywords:** head and neck cancer, drug repurposing, genomics

## Abstract

**Background/Objectives:** Head and neck cancer (HNC) represents the seventh most common cancer diagnosis globally, yet current treatments, including surgery, radiation, and immunotherapy, have shown limited improvement in outcomes. Drug repurposing offers a cost-effective strategy to identify new therapeutic options by leveraging existing medications with known safety profiles. Within this study, we developed the **GARD** pipeline (**G**enomic **A**lteration-based **R**epurposing for **D**rugs), designed to uncover repurposing candidates for HNC using genomic and network-based approaches. **Methods**: GARD integrates multi-omics data from The Cancer Genome Atlas (TCGA), including copy number variation (CNV) and somatic mutations (SOM). The cohort was stratified by human papillomavirus (HPV) status. Risk-associated genes were identified and then expanded via high-confidence protein–protein interaction (PPI) networks. Top candidate genes were filtered through comprehensive analysis of publicly available literature data in PubMed using LLMs to validate the relationship between the identified genes and HNC. The top risk genes and their network-expanded neighbors were mapped against DrugBank, and through statistical significance testing and literature validation, established significant drug–gene associations. **Results**: Significant genes associated with HNC, inferred by genomics alteration, were identified across HPV-positive and HPV-negative subgroups, such as PIK3CA, SOX2, TP53, EIF4G1, TLR7, CLDN1, PRKCI, and EPHA2. Further expansion through the PPI network identified other targetable genes such as EGFR, ERBB2, and the FGFRs. Literature-based validation efforts ensured confidence in the gene–disease association. Drug–gene mapping revealed candidates spanning those already in clinical trials for HNC (e.g., Afatinib, Cabozantinib, Dasatinib, Brigatinib, Lenvatinib, Capivasertib, and Erdafitinib) and emerging or repurposing candidates (Amuvatinib, XL765 (Voxtalisib), Golotimod, Artenimol, Quercetin, and Acetylsalicylic Acid), offering opportunities for precision repurposing. **Conclusions**: The GARD pipeline demonstrates a genomics-driven, network-informed framework for systematic drug repurposing in HNC. HPV stratification enhances precision, literature-based validation strengthens confidence, and integrated drug mapping enables refinement of existing therapies and discovery of novel candidates for personalized treatment strategies. **Code Availability**: The full implementation of the GARD pipeline, including preprocessing scripts, statistical analysis modules, and visualization tools, is publicly available on GitHub.

## Simple Summary

Head and neck cancer (HNC) is among the most prevalent and challenging cancers worldwide. Developing new drugs is expensive and time consuming, so this study explored a faster, cost-effective approach utilizing existing medications with established safety profiles: drug repurposing. We developed the **GARD**pipeline (**G**enomic **A**lteration-based **R**epurposing for **D**rugs), which utilizes large-scale genomic data from The Cancer Genome Atlas (TCGA) to identify key genomic changes in HNC. These genes are expanded through protein–protein interaction networks to capture related pathways and then validated using evidence from thousands of PubMed articles extracted by large language model (LLM) tools. Finally, validated genes are matched with drugs using the DrugBank database. This approach uncovered both known cancer drugs and promising new candidates. These included targeted therapies such as Fostamatinib, Nintedanib, Brigatinib, Regorafenib, and Lenvatinib, as well as emerging compounds like Artenimol, Quercetin, and Acetylsalicylic Acid (Aspirin). Through a combination of genomic analysis, network expansion, and literature validation, the GARD pipeline offers a powerful way to accelerate personalized cancer treatments while reducing cost and development time.

## 1. Introduction

Head and neck cancer (HNC) includes a diverse group of malignancies arising in the oral cavity, pharynx, hypopharynx, larynx, nasal cavity, and other regions of the head and neck, ranking as the seventh most common cancer globally [1]. Common treatments include surgical resection followed by radiotherapy and chemotherapy [2]. The medications/drugs in use today to treat HNC are still limited. Targeted therapies such as kinase inhibitors and immunotherapies have shown promise in other cancers and have been used in HNC recently [3]. Despite these advances, the treatment of HNC is still challenging due to the heterogeneity and novel treatments still have a hard time making it to market [2]. Additionally, a crucial factor to consider in HNC is HPV status, which significantly influences tumor biology, treatment response, and overall prognosis. Stratifying patients by HPV status enables more precise identification of genomic alterations and improves the relevance of therapeutic candidates for HPV-positive and HPV-negative subtypes [2,4].

Identifying more targeted treatments could significantly improve outcomes for patients with HNC. One promising and cost-effective strategy is drug repurposing, which involves finding new therapeutic uses for existing medications [5]. This strategy offers a cost-effective and time-efficient alternative to traditional drug development, as repurposed drugs have already undergone extensive safety and pharmacokinetic testing [6]. Computational repurposing approaches, which leverage large-scale biomedical datasets to systematically uncover mechanism-based drug–disease relationships, have shown potential [5,6,7,8]. Genomic-based computational methods include integration of genomic, transcriptomic, proteomic, and pharmacological data to identify drugs with potential efficacy against molecular targets. By harnessing publicly available multi-omics data, researchers can expand therapeutic options through the repositioning of approved or investigational compounds, ultimately advancing precision oncology [9,10].

In cancer research, genomic-based computational drug repurposing has already led to promising therapeutic advances. Recent advancements in available large-scale cancer genomics data have allowed for the investigation of cancers driving genetic therapeutic targets [11,12,13]. Cancer is driven by changes in the genome, and studying these changes in HNC can help identify key driver genes in tumor initiation and progression [2,14]. In this way, repurposed drug candidates that target key disease-related genes can be identified. The Cancer Genome Atlas (TCGA) and Cancer Cell Line Encyclopedia (CCLE) are some of the datasets that serve as sources for cancer-related genes. They provide information about copy number variations (CNVs), somatic mutations (SOMs), clinical data, and drug screens on cell lines [12,15,16].

The TCGA dataset serves as one of the main data sources for cancer-related genes. Existing TCGA-based drug repurposing varied in approaches and targets. Many studies focused on identifying therapeutic candidates in varied cancers, through differential gene expression towards patient outcomes such as binary cancer indicators, survival using linear models, or Cox regression models to identify genomic features associated with patient outcomes [17,18,19,20,21,22]. These and other studies focused around genomic-based analysis to identify underlying mechanisms and potential for use in treatment [20,23,24,25]. The use of CNVs or mutations from cancer genomics data for drug repurposing is less prevalent. Within HNC specifically, some studies narrowed in on specific medications or niche conditions [26]. Among these, one study maintained a broad approach to HNC alongside network expansion, as in this study, but did not incorporate HPV stratification and chose other methods of validation to land on very few specific repurposing candidates [19]. Some included HPV stratification, but were focused specifically on known or immediate alterations within the HNC genomic landscape and did not incorporate any network expansion [27].

Based on previous repurposing studies, a major limitation of current approaches lies in the lack of rigorous filtering and validation to prioritize a high-confidence set of drugs for clinical application [5]. Current works were able to identify possible repurposing candidates, but utilized one data source and needed larger manual review for identifying top candidates [5,15]. Additionally, many previous studies were effective in drug repurposing but had limited stratification of HPV analysis, or focused on survival-associated genes rather than a comprehensive genomic analysis, and only a few incorporated the use of robust validation steps to confirm candidate relevance and narrow repurposing candidates. The incorporation of network expansion to broadly identify possible HNC-targetable genes alongside HPV-stratified TCGA data with external validation provides a unique opportunity and approach to producing repurposing candidates. This study works to fill these gaps and presents a repurposing pipeline to produce a validated set of drug repurposing candidates from HPV-stratified TCGA genomics data.

This study introduces the **GARD** pipeline (**G**enomic **A**lteration-based **R**epurposing for **D**rugs) which integrates HPV stratification with large scale multi-omics to identify specific key disease related genes within HPV +/− patients. These genes are expanded using high confidence interactions with other proteins to ensure relevant biological pathways are included, and rigorously validated using thousands of peer-reviewed articles from PubMed. The use of PubMed literature in drug repurposing has had promising results, and the use of such methodologies as an exterior validatory method has the ability to ensure viable repurposing candidates are identified [28]. Lastly, the identification of medications is completed using DrugBank, where known drug–gene combinations are curated, and thorough empirical validation is performed. The results from our drug repurposing pipeline, GARD, confirmed the validity of its performance through the identification of both established and novel therapeutic candidates for HNC. In this study, we demonstrate that genomics-based computational drug repurposing can provide a scalable and precise framework for discovering new treatment options tailored to molecular subtypes, such as HPV-positive and HPV-negative HNC. These identified medications can serve as hypotheses of treatment avenues in HNC that warrant further exploration through clinical and experimental studies, with the goal of translating them into effective patient treatments. Given the heterogeneity of many cancer types and the growing availability of genomic datasets, the presented pipeline, GARD, holds promise for broader application across other malignancies and even non-cancer diseases, potentially accelerating the discovery of novel therapeutics, reducing development costs, and expanding precision medicine.

The GARD pipeline, including preprocessing scripts, statistical analysis modules, and visualization tools, is publicly available on GitHub at: https://github.com/pvtanike/Genomic-Landscape-Based-Drug-Repurposing.git (accessed on 17 February 2026).

## 2. Materials and Methods

### 2.1. GARD Pipeline Overview

To identify drug repurposing candidates for HNC, we developed the GARD pipeline. This framework integrates multiple publicly available biomedical resources to combine genomic insights with pharmacological and publicly available literature data for the identification of drug repurposing candidates in HNC.

First, at its core, GARD leverages TCGA, which provides multi-omics datasets, including Copy Number Variation (CNV) and Somatic Mutation (SOM) profiles with associated clinical annotations. The datasets were analyzed to produce high-confidence risk genes associated with HNC. All data was used alongside results of previous work to stratify patients by human papillomavirus (HPV) status [13]. This stratification enables subtype-specific analysis and improves therapeutic relevance [12].

Second, to capture pathway-level context, GARD expands high-confidence risk genes through STRING protein–protein interaction (PPI) networks, incorporating immediate neighbors with strong biological connectivity [29]. These genes are validated through external data sources of publicly available literature data within PubMed, as literature-based repurposing efforts have shown effectiveness [28,30].

Third, after extraction and cleaning, the literature data was parsed by the LLM model, Google GEMMA, to identify genes associated with HNC and provide peer-reviewed evidence as support for the gene association with HNC and filter the identified gene list for drug–gene associations [31].

Fourth, to connect these validated genetic findings with potential therapeutic agents, GARD incorporates curated drug–gene data from DrugBank [32]. To ensure that the drugs identified have a significant relationship with target genes identified either directly from CNV/mutation analysis or as the protein neighbor genes, statistical enrichment-based tests were performed for robustness.

**Figure 1:**
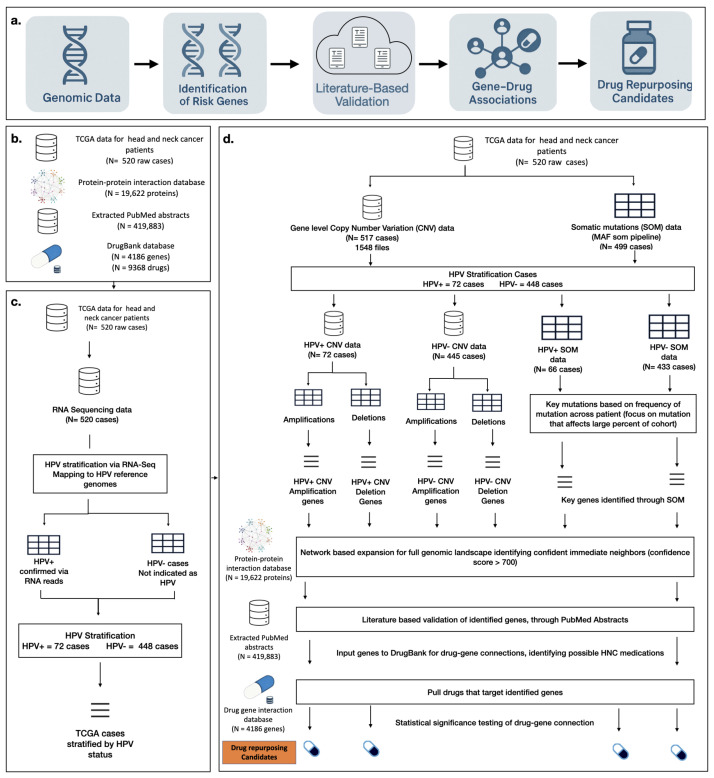
Overview of the GARD analytical pipeline for identifying drug repurposing candidates in HNC. (**a**) Illustrates the overall workflow, which begins with acquiring key genomic data, followed by identifying high-confidence genes, validating genes through literature-based analysis, and mapping them to drugs through curated gene–drug associations, ultimately yielding candidate medications for repurposing. Each section of this workflow is further broken down in the following section of the diagram, which begins with (**b**) input data sources, which include multi-omics data from TCGA HNC cohort (520 raw cases curated from Case ids from Nulton et al. [13]), the STRING PPI network comprising approximately 19,622 human proteins, extracted PubMed abstracts totaling 419,883 raw abstracts associated with HNC, and the DrugBank drug–gene interaction database containing 9368 drugs and 4186 protein targets [12,29,32]. Next, (**c**) HPV stratification is based on work completed by Nulton et al. [13], in which RNA-Seq data was used by extracting unmapped reads and aligning them to a reference panel of 171 HPV genomes, confirming HPV positivity primarily for HPV16, the predominant genotype in HNC, and classifying cases into HPV-positive (*n* = 72) and HPV-negative (*n* = 448) subgroups. Finally, (**d**) the drug repurposing pipeline integrates genomic alteration analysis, where copy number variation (CNV) and somatic mutation (SOM) data are processed to identify significant amplifications, deletions, and mutations across HPV-stratified cohorts. High-confidence risk genes are determined through statistical significance testing using binomial and permutation-based approaches with false discovery rate correction, then expanded through high-confidence PPI networks (STRING score ≥ 700) to capture pathway-level relevance. The genes were then validated through a literature-based validation step, leveraging publicly available sources (e.g., PubMed) to confirm drug–gene associations using recent research evidence. These validated gene sets are cross-referenced with DrugBank to identify drugs targeting either direct risk genes or their immediate network neighbors, and candidate drugs are determined using enrichment-based statistical tests, including hypergeometric and permutation analyses, ensuring significant drug–gene connections and resulting in a set of both approved therapies and novel repurposing opportunities.

**Figure 2:**
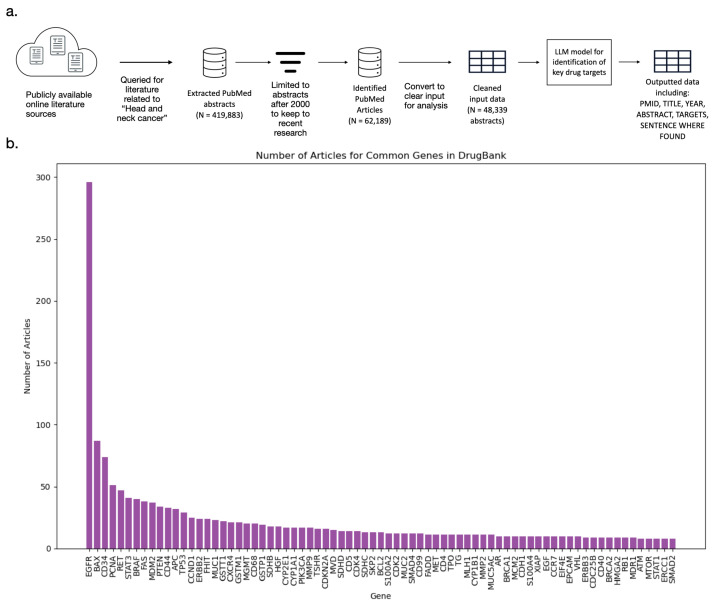
Literature-Based Validation Pipeline and Identified Gene Targets for HNC. (**a**) The validation pipeline workflow. The diagram illustrates a multi-step workflow designed to complement genomic analyses by leveraging published research. The process begins with querying PubMed for large-scale acquisition of biomedical abstracts related to HNC using NCBI’s E-utilities, followed by filtering to include only recent literature (published after the year 2000). The curated data is then formatted into structured tables to optimize interpretation by a large language model (LLM). Using Google GEMMA, abstracts are parsed to extract key therapeutic targets and supporting evidence. The final output integrates gene targets, source references, and associated drugs. (**b**) Top 75 gene targets ranked by literature support. Genes are displayed in descending order based on the number of PubMed articles referencing their association with HNC. Highly studied targets such as EGFR, TP53, PIK3CA, and CDKN2A dominate the top ranks, while emerging candidates with fewer publications highlight novel therapeutic opportunities.

Together, these resources form the foundation of a robust analytical framework designed to uncover clinically relevant drug repurposing opportunities and establish a scalable methodology applicable to other diseases. The approach centers on three key components: (1) extraction and preprocessing of genomic data, (2) analysis, identification, and validation of HNC-specific genotypic features, and (3) connecting with DrugBank to pinpoint viable drug repurposing opportunities. A clear diagram of the pipeline can be seen in Figure 1. A breakdown of the literature validation pipeline is available in Figure 2a.

### 2.2. TCGA Data

The key genomic data was sourced from TCGA, a comprehensive and collaborative initiative between the National Cancer Institute (NCI) and the National Human Genome Research Institute (NHGRI): https://www.cancer.gov/tcga (accessed on 13 November 2025) [12]. For this study, the TCGA HNC cohort corresponding to case IDs from the Nulton et al. study was utilized, allowing for HPV stratification. The final cohort included 520 cases, which were further refined based on the availability within each data category: copy number variation (CNV), and somatic mutation (SOM) data [12,13]. All data was accessed through the Genomic Data Commons (GDC) Data Portal. This study utilized open-access data, which includes de-identified clinical information, gene-level copy number variation (CNV) and somatic mutation profiles (SOM).

### 2.3. HPV Stratification

HPV serves as an influential factor in the diagnosis and treatment of HNC [1,2,33,34]. Its importance within the GARD pipeline was no different. HPV stratification was performed using results from Nulton et al., who demonstrated that RNA-Seq data can be used effectively to determine HPV status in HNC [13]. The majority of HPV-positive cases were HPV16, which was the primary genotype analyzed due to its prevalence and clinical relevance. Other HPV types were detected with low frequency. The Nulton et al. study analyzed 520 TCGA HNC cases and identified 72 HPV-positive patients [13].

### 2.4. Genomic Amplifications and Deletions

After stratification on HPV status, it was possible to utilize the data to identify key genomic components of HNC. Significant CNV amplifications and deletions were identified through statistical testing and GISTIC-like scoring, then used to highlight key genomic drivers and expanded to their high-confidence protein interaction neighbors for downstream therapeutic targeting. TCGA provides gene-level copy number variation (CNV) data, which quantifies changes in the number of copies of specific genes across tumor samples. Deviations from this baseline, either deletions (CNV < 2) or amplifications (CNV > 2), can indicate genomic alterations that are possibly associated with the cancer or disease being studied.

Based on the categorization from Nulton et al., the key genetic mutations were categorized into HPV+ and HPV- cohorts. Within the extracted HNC TCGA cohort, about 517 cases had cleaned CNV data. Within the available data for each sample, 58,645 available genes were analyzed and provided chromosome number, start and end position, and copy number value [12,35,36].

After identifying a gene as being amplified or deleted, it was important to determine whether the mutation was random or of significant prevalence across the entire disease landscape. In order to first filter to significantly altered regions for HNC, a significance test for high-level amplifications and deletions was calculated. High-level amplifications were considered as those with a CNV value greater than 4. Along the same lines, high-level deletions were considered as those equaling 0 [37]. To determine the significance of the mutation, the counts of high-level amplification or deletion were utilized to assess whether their occurrence across samples exceeded what would be expected by chance.

A binomial test was conducted where the significant mutation count per gene is the number of successes across all files. This approach enabled the derivation of a *p*-value to assess whether the CNV frequency in each gene exceeded what would be expected by chance, given the cohort’s overall genomic instability. The calculation of this value was:

P(X≥x)=∑k=xnnkpk(1−p)n−k

where *n* is the total number of samples in the cohort, *x* is the number of samples with high-level CNV amplification (CNV copy number >4) or deletion (CNV <1) events, and *p* is the genome-wide background CNV rate calculated as $p_{\mathrm{null}}=\frac{\sum ⊮({\mathrm{CNV}}_{i}>4)}{\mathrm{total}\;\mathrm{CNV}\;\mathrm{observations}}$ for amplifications, and  $p_{\mathrm{null}}=\frac{\sum ⊮({\mathrm{CNV}}_{i}<1)}{\mathrm{total}\;\mathrm{CNV}\;\mathrm{observations}}$ for deletions. This represented the proportion of all CNV measurements across all genes and samples that exceeded the high-level threshold. This approach tested each gene against the observed cohort-specific genomic instability.

Alongside this, an empirical null distribution was derived through permutation testing. For each 1000 permutations (selected to balance computational burden and significance), *n* CNV values were randomly sampled (with replacement) from the pooled set of CNV observations, and the count of sampled values exceeding the threshold (CNV > 4, CNV < 1) was recorded to build a null distribution. The empirical *p*-value was calculated by comparing the observed count to this null distribution. This was then divided by the number of permutations of the null distribution (*n* = 1000) to derive an empirical *p*-value. The empirical *p*-value was calculated as:

$$p_{\mathrm{emprical}}=\frac{1+\sum_{i=1}^{1000}⊮\left({\mathrm{count}}_{i}\geq x_{{\mathrm{obs}}_{i}}\right)}{1001}$$

where $x_{\mathrm{obs}}$ is the observed number of samples with high-level CNV events for the gene, ${\mathrm{count}}_{i}$ is the simulated count of high-level events in permutation *i*, ⊮ is an indicator function that equals 1 when the simulated count equals or exceeds the observed count, and the “+1” in both numerator and denominator prevents *p*-values of exactly zero while maintaining proper probability bounds. The two *p*-value calculations were conducted across many genes, and each test had its own chance of producing false positives. As such, a false discovery rate (FDR) adjustment was necessary. The Benjamini–Hochberg correction was applied for this study [38].

To further filter key mutations, a methodology was developed based on the methodology within the GISTIC software (GISITC 2.0) [37]. The key aspect is that mutations are assigned a score that considers both amplitude and frequency. Amplification values are accumulated as log2 of the CNV value minus 1 to adjust for the diploid baseline. This helped to center the data around 2 or a diploid CNV count. A cap was placed on CNV values (CNV = 7) to ensure that outliers do not skew values and reduce visibility of genes with lower CNV amplitude but higher frequency. Deletion values were accumulated as +1 for minimal deletions (CNV = 1) and +2 for significant deletions (CNV = 0) to track the amplitude of CNV alteration. The frequency of significant mutations was calculated by tracking the number of times a gene was significantly amplified (CNV > 4) or deleted (CNV < 1). For amplification calculations, the score was calculated as the average log2-adjusted amplification value (amplitude) multiplied by the frequency percentage (prevalence) of high-level mutations. For deletions, the GISTIC-like score was calculated as the average absolute deletion intensity (amplitude) multiplied by the frequency percentage (prevalence) of significant deletion (CNV = 0) [37].

Significant genes were first filtered to those with significant mutations according to the FDR-adjusted *p*-values in both the binomial and empirical significance tests. To ensure robustness and control false discoveries, we implemented a conservative dual-testing approach requiring candidates to pass both parametric and non-parametric significance tests. To further refine candidate selection, the distributions of GISTIC-like scores for these genes were evaluated to assess the strength of copy number alterations. Rather than applying arbitrary fixed cutoffs, we employed a distribution-based approach where thresholds were determined empirically from observed data patterns. For each analysis stratum (HPV-positive/HPV-negative × amplification/deletion), we visualized score distributions using histograms, examined distribution statistics (mean, percentiles), and identified natural breaks where scores transitioned from the bulk distribution to the upper tail. The selected parameters proved ideal for identifying key risk genes. The final cutoffs were implemented to systematically identify top-priority genes within each subgroup. By integrating statistical significance with genomic alteration burden, this process prioritized a focused set of high-confidence genes most relevant to HNC. These key genes could serve as potential targets for medications and, in turn, identify possible repurposing candidates [5,39].

### 2.5. Somatic Mutations

In addition to the copy number variations (CNVs), somatic mutation (SOM) data was utilized. Somatic mutation (SOM) data was analyzed using length-normalized mutation burdens, statistical significance testing, and empirical permutation methods to identify key mutated genes in HNC, which were then expanded through high-confidence protein–protein interaction neighbors to support downstream therapeutic targeting. Somatic mutations were identified using cohort-level MAF analysis files from TCGA [13]. This identified cohort included information on a total of 16,477 mutations for the 499 cleaned cases available within the data. This data was stratified by the HPV status as described above, leading to 66 HPV-positive cases and 433 HPV-negative cases. After stratifying the data by HPV status, key genes were tracked by monitoring their mutations, using a method similar to the CNV tracking approach described in the above section.

The mutations were filtered to focus on non-synonymous mutation types, as synonymous mutations rarely impact protein function [40]. Gene lengths were extracted from GENCODE v48 annotations and used to normalize mutation counts, as longer genes have a greater opportunity to accumulate mutations by random chance simply due to larger mutational target size [41]. Each gene in the available data was analyzed to count the number of times a mutation occurs in a gene, as well as the unique number of patients harboring a mutation for the gene. Producing a mutation count and cohort frequency that were normalized by gene length.

A binomial test was then conducted to measure the enrichment of each mutation within the cohort and the significance of the mutation. Through the mutation-based analysis, the mutational burden of each gene within the cohort could be directly measured. This test observed whether the mutation count in a gene significantly exceeds the expected count based on its genomic target size. The test assumes the observed mutation count as the number of successes and the total number of mutations across the cohort as the trial size. The test statistic was calculated as:

P(X≥x)=∑k=xNNkpk(1−p)N−k

where *N* is the total number of mutations observed in the cohort (including both synonymous and non-synonymous mutations to establish an unbiased background rate), *x* is the observed number of non-synonymous mutations in the gene, and *p* is the gene-specific probability calculated as the $p=\frac{L_{\mathrm{gene}}}{\sum_{j=1}^{g}L_{j}}$ gene, where $L_{gene}$ is the coding sequence (CDS) length of the gene, and the denominator represents the sum of CDS lengths across all protein-coding genes in the reference genome. This length-based analysis accounts for the fact that longer genes present larger mutational target spaces and are expected to accumulate more mutations by chance. The test directly measures whether the mutational burden in each gene exceeds expectations given its genomic target size.

As performed in the above section, an empirical significance test was conducted as well. For each gene, a multinomial null distribution is constructed based on the mutation cohort, the probability of gene mutation is based on gene length calculated as gene length/sum of gene lengths. The null was constructed for each gene to be the size of the number of simulations (*n* = 10,000). The number of simulations was increased to account for the sparse nature of mutation events in somatic data and balanced with computational capabilities. A multinomial distribution was used to account for different mutation probabilities based on gene lengths.

$$\mathrm{null}\;\mathrm{distribution}∼\mathrm{Multinomial}(N_{\mathrm{total}},p)$$

where $N_{total}$ is the total number of mutations observed across the cohort and p is the gene-specific probabilities, with $p_{j}=\frac{L_{j}}{\sum_{k=1}^{g}L_{k}}$ based on the CDS length $L_{j}$ of each gene. The multinomial distribution enables simultaneous simulation of mutation counts across all genes while preserving the constraint that total mutations remain constant and respecting differential mutational target sizes. The empirical *p*-value was calculated as:

$$p_{\mathrm{empirical}}=\frac{1+\sum_{i=1}^{10000}⊮\left({\mathrm{count}}_{i}\geq x_{\mathrm{obs}}\right)}{10001}$$

where $x_{\mathrm{obs}}$ is the observed mutation count in the gene and ⊮ is an indicator function that equals 1 when the simulated count equals or exceeds the observed count. As before, both binomial and empirical *p*-values were FDR corrected using the Benjamini–Hochberg procedure. Genes with FDR-adjusted *p*-values < 0.05 in both tests were retained as significantly mutated, ensuring stringent control over false discoveries while maintaining adequate statistical power [38]. These statistical tests were utilized to remove background noise and filter to significantly mutated genes in the studied cohorts. From here, candidates were identified using similar methodologies to CNV analysis. A composite mutation score was calculated for each gene as:

MutationScore=NormalizedCount×FrequencyPercentage

where Normalized Count represents the mutation burden per gene adjusted for coding sequence length ($\frac{\mathrm{mutation}\;\mathrm{count}}{L_{\mathrm{gene}}}$), and frequency percentage is the proportion of patients harboring mutations in the gene. This scoring framework balances mutation amplitude (burden per gene) with cohort prevalence (frequency across patients), ensuring that both recurrent and high-impact mutations are captured.

As with CNV analysis, genes were retained only if they passed both statistical tests, ensuring robustness. Following statistical filtering, top-priority genes were selected by applying cutoffs to the mutation score distribution, ensuring retention of genes with the highest mutational burden. To characterize mutation burden patterns, mutation score distributions were visualized and evaluated to identify natural inflection points marking separation between the bulk distribution and the upper-tail region enriched for biologically meaningful mutational events. The selected values provided balance between stringent filtering and biological coverage for identifying driver genes. These cutoffs were incorporated to systematically identify high-priority mutated genes within each analysis subgroup. Through integration of statistical significance with genomic alteration burden, this process prioritized a focused set of high-confidence genes that could be used to identify treatments for HNC and possible repurposing candidates.

### 2.6. STRING Protein–Protein Interaction (PPI) Network

Through the above methods, it was possible to identify key drug targets directly related to HNC. However, beyond the primary key genes directly implicated in HNC, their immediate network neighbors, genes connected either upstream or downstream in the relevant biological pathways, were also included in the analysis. This approach allowed for the consideration of both identified HNC direct risk genes and those with a single-degree interaction in the pathway context. Identifying these neighbors helps to account for other key genes that could potentially serve as drug targets [5,42]. Within the GARD pipeline, the STRING PPI network was utilized [29]. For this study, the STRING database was filtered to focus on homo sapiens (human) gene interactions and pathways (Version 12 accessed on 29 July 2025).

The direct risk genes identified through CNV and SOM mutations analysis were inputted to the STRING PPI to identify immediate neighbors of the genes. Once immediate neighbors were identified for the key genomic mutations, the top connections were limited based on their confidence score. Based on the STRING db documentation, a cutoff of 700, representing high-confidence connections while providing broader biological coverage than more restrictive thresholds, was used to keep top immediate neighbors [29]. The culminated top connected genes and key mutations of HNC were then utilized with the drug gene interaction database to identify medications that interact with these key targets.

### 2.7. LLM-Based Literature Extraction for Gene Validation

The literature-based validation pipeline was designed to complement genomic analyses by leveraging published research as an independent validation layer to verify and reinforce identified gene–HNC associations in drug candidates, ultimately identifying validated drug repurposing opportunities. The validation dataset was derived from publicly available resources in PubMed, a comprehensive repository including peer-reviewed biomedical and life sciences literature. The process begins with the large-scale acquisition of PubMed biomedical abstracts using NCBI’s E-utilities, a suite of tools designed for searching, retrieving, and analyzing PubMed records [30,43]. A query of “Head and Neck cancer” was provided to PubMed to extract all relevant literature for HNC key target identification (accessed on 6 August 2025). This query leveraged PubMed’s MeSH indexing system, which automatically maps search terms to controlled vocabulary, ensuring comprehensive capture of all relevant anatomic subtypes [44]. The query returned 419,883 abstracts, including titles, publication years, and abstracts. The dataset was then cleaned to remove incomplete or missing abstracts and filtered to include only studies published within the past 25 years (i.e., after the year 2000), ensuring relevance and contemporary scientific context. After cleaning and filtering, a total of 48,339 unique abstracts were retained for analysis.

The data was then structured for input to the model, which included essential metadata such as Pubmed ID (PMID), title, publication year, and abstract text, as shown in Table 1. After compiling and formatting the data that included abstracts related to HNC, the LLM Google GEMMA was utilized. The Google GEMMA 2B instruction version was utilized (gemma-2b-it). Google GEMMA has proven ability to interpret text and extract key information [31]. Due to the computational intensity of processing such a large dataset, the University of North Carolina at Chapel Hill (UNC) Longleaf High-Performance Computing Cluster was utilized to ensure efficiency and scalability.

The LLM was prompted to identify key therapeutic targets related to HNC and extract supporting evidence by locating the specific sentence within each abstract where the target was mentioned. The output structure, illustrated in Table 2, included the identified gene, the evidence sentence, PMID, and any associated drugs, enabling transparent verification and efficient downstream analysis. This output format was intentionally designed to streamline manual review by enabling the rapid inspection of gene–disease associations alongside their supporting sentences and source articles. This aided in ensuring appropriate validation occurred for associations identified through CNV and SOM analysis. The output structure facilitated this quick manual review. An overview of this design analysis can be seen in Figure 2a.

### 2.8. Drug–Gene Connection for Drugs in DrugBank

After identifying immediate neighbors through high-confidence STRING PPIs and validating both these neighbors and the top risk genes through literature-based analysis, the fully validated gene set was queried against DrugBank to determine drug–gene associations [29,32]. This ensured that both direct risk genes and their biologically relevant neighbors were incorporated, thereby broadening the landscape of potential repurposable drugs. We then assessed whether these validated genes corresponded to drug targets in DrugBank and evaluated the statistical significance of their drug–gene connections to identify therapeutically relevant agents and prioritize candidates for repurposing in HNC. DrugBank is a curated database, providing detailed annotations on experimentally validated drug–gene interactions, including the mechanism of action (e.g., inhibitor, activator, modulator). It contains information on 9368 medications and 4186 genes totaling 23,136 drug–gene interactions (Version 6.0 accessed on 31 July 2025).

To assess the statistical significance of drug–gene connections, two complementary enrichment approaches were applied. First, a right-tailed hypergeometric test via cumulative binomial distribution was used to evaluate whether a given drug targeted more risk genes than expected by chance, given all druggable genes in DrugBank as the background of the test. The method quantifies the likelihood of seeing the observed or more overlap in gene targets between the drug’s targets and risk genes, and to determine if this is more than expected by chance. Here, the null hypothesis stated that a drug’s target genes overlapped with the risk gene set randomly, while the alternative hypothesis indicated significant enrichment. The test statistic was calculated as:

P(X≥x)=∑i=xmin(K,n)KiM−Kn−iMn

where *M* is the total number of druggable genes in DrugBank (4186), *K* is the number of targets for a given drug, *n* is the number of risk genes (including PPI neighbors) present in DrugBank, and *x* is the observed overlap between the drug’s targets and the risk set. By normalizing for each drug’s total target count (*K*), this test inherently controls for medications with broad target profiles.

Second, an empirical permutation test was conducted to strengthen confidence in the results. In this test, random gene sets of equal size to the risk set were repeatedly sampled (100,000 permutations) from the background universe of drug targetable genes (all available genes within DrugBank). The large number of permutations was used here to ensure that the drug–gene associations were significant and ensure maximum precision with the computational power available. For each permutation, the overlap with drug targets was calculated. The empirical *p*-value was then defined as the proportion of permutations with an overlap greater than or equal to the observed overlap. This distribution-based approach controlled for potential biases in gene–drug network structure. The empirical *p*-value was then defined as:

$$p_{\mathrm{empirical}}=\frac{1+\sum_{i=1}^{10000}⊮\cdot \left({\mathrm{overlap}}_{i}\geq x_{\mathrm{obs}}\right)}{100001}$$

where $x_{\mathrm{obs}}$ is the observed overlap and the indicator function ⊮· equals 1 when the permuted overlap equals or exceeds the observed overlap. This empirical validation ensures that observed enrichment is not due to highly connected genes. This distribution-free approach provides robustness against deviations from hypergeometric assumptions and accounts for heterogeneity in drug target set sizes.

Both hypergeometric and empirical *p*-values were FDR corrected for using the Benjamini–Hochberg procedure. Drugs with FDR-adjusted *p*-values < 0.05 in both tests were retained as significant candidates [38]. This approach enabled the filtering of medications to identify those with significant drug–gene interactions. In conjunction with a set of genes identified through CNV and SOM analyses, as well as their immediate neighbors, these drugs demonstrate a strong association with HNC, with only those meeting both statistical enrichment and the literature validation of genes advancing as drug repurposing candidates. The medications hold therapeutic potential; those not originally indicated for HNC could serve as repurposing candidates.

## 3. Results

### 3.1. Overview of the GARD Pipeline

We developed the GARD pipeline to perform a comprehensive analysis of HNC using genomic data from TCGA, stratified by human papillomavirus (HPV) status. Through GARD’s multi-step framework, integrating copy number variation (CNV), somatic mutation (SOM) profiles, PPI networks, and curated drug–gene associations from DrugBank, the pipeline identifies both established and novel genetic targets with therapeutic potential. An overview of the pipeline can be seen in Figure 1.

Genomic alterations were categorized into three major types: copy number amplifications, copy number deletions, and somatic mutations. Each category was analyzed independently to identify statistically significant gene-level changes associated with disease progression. High-confidence associated genes were identified using a combination of binomial and empirical permutation testing, with FDR-adjusted *p*-values. This allowed for the removal of background noise and filtering for significantly mutated genes. The top significant genes were further filtered based on composite scores developed based on a combination of mutation amplitude (strength of mutation) and frequency (number of cases affected). These genes were then expanded through PPI networks in the STRING database using a PPI combined score > 700 as the cutoff for high-confidence interactions in PPI. This allows for the inclusion of immediate neighbors that may play biologically relevant roles in HNC pathogenesis [29].

After that, a key step within GARD of literature-based validation was performed for the above genes using PubMed abstracts parsed by the Google GEMMA LLM, which strengthens confidence in gene–disease associations and prioritizes biologically relevant targets [28,31]. Through large-scale analysis of PubMed abstracts, a comprehensive set of 6077 unique genes associated with HNC was identified. Figure 2b illustrates the top 75 genes, sorted by article frequency, highlighting the most extensively studied targets in HNC. The identified genes include well-established oncogenic drivers such as EGFR, TP53, PIK3CA, and CDKN2A, which are frequently implicated in tumor progression and therapeutic resistance. These findings confirm the robustness of the pipeline in capturing both key cancer genes and less-studied targets with potential biological significance. This literature-based validation step strengthens confidence in the genomic findings by providing independent evidence from peer-reviewed research.

Genes were then mapped to drug–gene associations using DrugBank, identifying both direct drug targets and network-derived candidates across HPV-positive and HPV-negative cohorts. To assess the statistical significance of drug–gene connections, two complementary enrichment approaches were applied: a right-tailed hypergeometric test to evaluate whether a given drug targeted more risk genes than expected by chance, and an empirical permutation test randomly sampling genes from the DrugBank universe compared observed overlaps with risk genes to that of the null distribution. DrugBank provided crucial data regarding drug–gene interactions and information on medications, including their toxicity, allowing for selection of ideal repurposing candidates. This integrative approach revealed distinct molecular vulnerabilities within each subgroup and prioritized medications with strong mechanistic relevance to the identified gene sets. The following sections incorporate these methods to identify results and repurposing candidates.

Table 3 summarizes the number of high-risk genes identified through CNV and somatic mutation analyses, their connected immediate neighbors, and the corresponding significant medications uncovered through enrichment-based filtering.

### 3.2. HPV-Positive Investigation

Analysis of HPV-positive samples using CNV and somatic mutation data identified distinct genomic alterations associated with head and neck cancer. Table 3 summarizes the cohort-level results, showing that the HPV-positive CNV amplification analysis yielded 169 high-risk genes, of which eight were available as drug targets, and led to 1874 immediate neighbors totaling 627 targetable genes, resulting in 143 significant medications. The CNV deletion analysis identified 274 high-risk genes, of which 20 were available as drug targets, and led to 1518 immediate neighbors totaling 458 targetable genes, resulting in 42 significant medications. Somatic mutation analysis revealed six high-risk genes, of which one was available as a drug target (PIK3CA) and led to 338 immediate protein neighbors totaling 135 targetable genes, resulting in 90 significant medications from DrugBank. Overall, directly targeting the identified risk genes and their protein neighbors led to a total of 168 drugs to be repurposed, which is still relatively large. Of these 168 drugs, 14 were identified by direct connection to HNC risk genes, and 163 were identified through indirect connection between disease and drugs via protein–protein interactions. There were nine drugs identified from both procedures: Buparlisib, CH-5132799, Cladribine, Copanlisib, Golotimod, NADH, TG-100801, Wortmannin, and XL765.

To further refine this candidate set, literature validation of the genes was applied. A total of five genes (Appendix A Table A1) were validated in the literature among the above 51 unique risk genes and found to be targetable or connected to targetable genes through PPI. Among the validated risk genes, somatic mutations identified PIK3CA; amplification analysis derived PIK3CA, RFC4, CLDN1 and SOX2; and deletions identified TLR7. From here, the 168 drugs were reduced to 90, of which 6 were found by directly targeting risk genes (shown in Appendix A Table A2) and 85 targeted the protein neighbors of identified risk genes through protein–protein interactions (shown in Appendix A Table A3). One medication was found to overlap between the two sets: XL765 (Voxtalisib).

**Figure 3:**
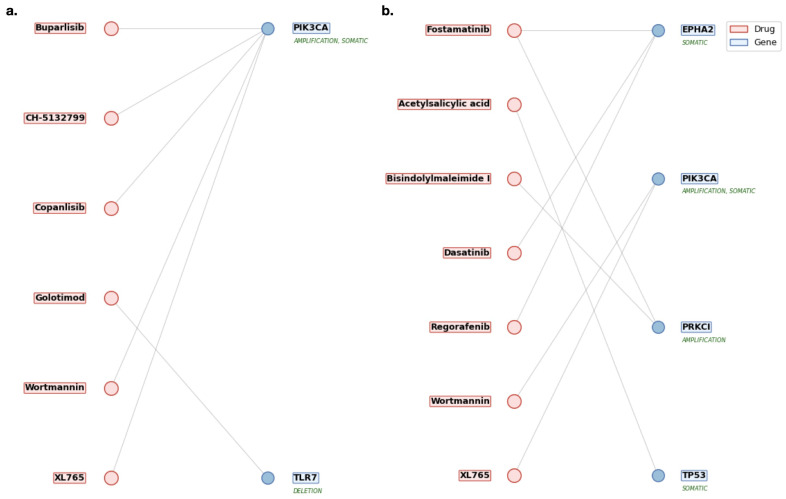
(**a**) Drug repurposing candidates targeting direct genomic risk genes in HPV-positive HNC. This figure presents medications identified through integrative analysis of copy number variations and somatic mutations in HPV-positive tumors. Each drug is linked to a direct genomic target, such as PIK3CA or TLR7, that was statistically validated as a high-confidence driver of disease. The listed candidates represent potential therapeutic agents for precision oncology in this distinct HNC subgroup. Candidates included CH-5132799, Golotimod, Buparlisib, and Copanlisib with strong mechanistic ties to HNC treatment. (**b**) Direct genomic targets identified in HPV-negative HNC from TCGA analysis. This figure presents a curated list of medications identified through integrative genomic analysis of HPV-negative HNC tumors using TCGA data. It highlights drugs that directly target high-confidence genomic risk genes uncovered via copy number variation (CNV) and somatic mutation profiling. These candidates, including agents such as Fostamatinib and Regorafenib, demonstrate therapeutic relevance by interacting with key oncogenic drivers like TP53, PIK3CA, and EPHA2, offering promising avenues for precision drug repurposing in HPV-negative HNC. These are linked to drug candidates of Acetylsalicylic acid, Regorafenib, Dasatinib, and Fostamatinib.

Identified risk genes were utilized with DrugBank to identify medications that directly target risk genes (direct targets) and develop significant drug–gene associations. Drugs directly targeting risk genes are illustrated in Figure 3a, highlighting key genes with existing therapeutic agents and potential candidates for drug repurposing (Table in Appendix A Table A2). Among direct targets, PIK3CA was associated with medications such as Wortmannin, XL765 (Voxtalisib), Buparlisib, Copanlisib, and CH-5132799, with the latter three identified as inhibitors. Buparlisib and Copanlisib have progressed to initial trials in combination regimens [45,46,47,48], CH-5132799 has shown potent antitumor activity in preclinical models, though it remains investigational [49], and XL765 (Voxtalisib) shows promising PI3K inhibition [50,51]. Wortmannin, despite its PI3K inhibition, is limited by poor selectivity and toxicity [52]. In contrast, TLR7 was linked to Golotimod, an immune-modulatory agent that completed Phase II trials for oral mucositis in HNC patients [53].

**Figure 4:**
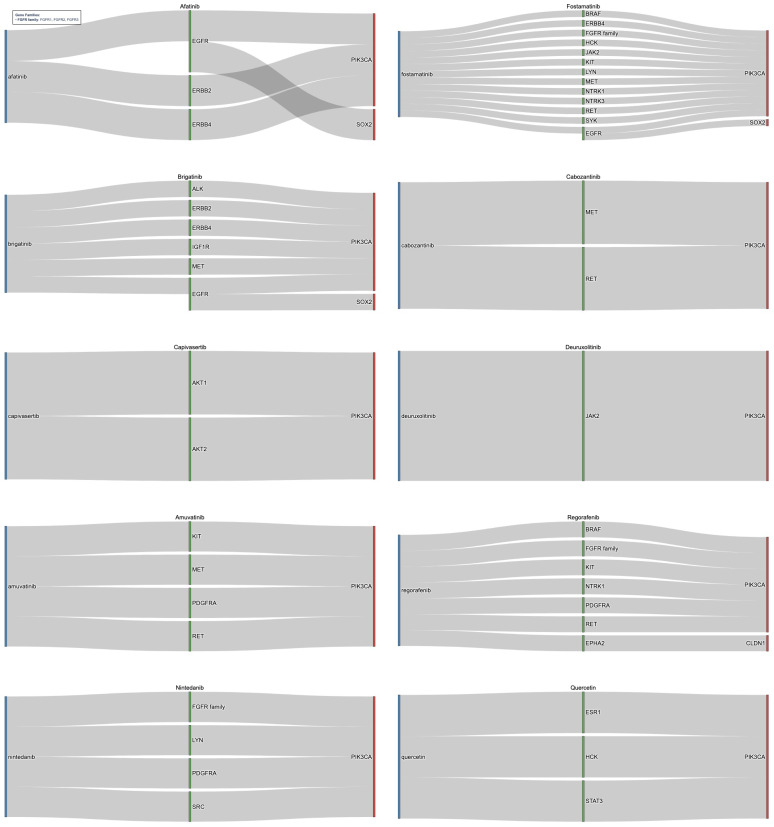
Sankey diagram for drug repurposing candidates target indirect genomic risk genes in HPV-positive HNC. This figure shows medications identified through network-based expansion of direct genomic targets using high-confidence PPI data. Each diagram shows the drug (blue), its gene target (green), and all connected risk genes (red), which were identified through CNV or SOM analysis, that are neighbors to the gene target. The drugs target immediate neighbor genes, such as EGFR, STAT3, and the FGFR family, which are functionally connected to core drivers of HPV-positive HNC and represent biologically relevant therapeutic opportunities. These were found through connections to key risk genes of PIK3CA, SOX2, and CLDN1. In this manner, kinase inhibitors such as Amuvatinib and Regorafenib, alongside medications such as Quercetin, were identified.

Furthermore, identified risk genes were expanded through high-confidence PPI networks, incorporating biologically relevant neighbors (indirect targets). Through validation of immediate neighbors and the direct risk genes they were connected to, the 263 unique druggable immediate neighbors originally identified were reduced to 33. Figure 4 illustrates the relationships between drugs, their gene targets, and high-risk genes for the top candidates identified by the pipeline. Top candidates were selected from the top 50 medications ranked by FDR-adjusted significance of drug–gene associations in HPV-positive HNC (full list in Appendix A Table A3). The medications target critical oncogenic pathways stemming from connections to risk genes of PIK3CA, SOX2, and CLDN1 underscoring their role in HNC [54,55,56,57]. Notable candidates included kinase inhibitors such as Afatinib, Fostamatinib, Brigatinib, Cabozantinib, Capivasertib, Deuruxolitinib, Amuvatinib, Regorafenib, and Nintedanib that work to target critical nodes including EGFR, MET, RET, KIT, PDGFRA, IGF1R, ALK, JAK2/3, AKT iso-forms, and the ERBB, FGFR, NTRK families.

Afatinib, an EGFR/ERBB inhibitor, showed efficacy in recurrent/metastatic HNC in trials such as LUX-Head & Neck [58,59], while Cabozantinib improved progression-free survival when combined with cetuximab or immune checkpoint inhibitors [60,61,62]. Fostamatinib stands out for its broad multi-kinase inhibition and Phase II evaluation in solid tumors, including HNC [63]. Capivasertib, a potent AKT inhibitor, selectively targets AKT1 and AKT2 and has demonstrated preclinical efficacy in HNC models, highlighting its potential to disrupt PI3K/AKT-driven oncogenesis [64,65,66]. Deuruxolitinib, a JAK2 inhibitor, represents an emerging candidate due to its ability to modulate the JAK/STAT pathway, which is frequently activated in HPV-positive tumors and associated with immune evasion and inflammation, making it a rational choice for repurposing [3,67]. Nintedanib has progressed to Phase II clinical trials in HNC, owing to its FGFR-targeting activity, addressing a key vulnerability in HPV-positive disease [68]. Brigatinib, originally approved for non-small cell lung cancer, inhibits ALK and EGFR signaling, suggesting strong potential for overcoming resistance mechanisms in HPV-positive HNC [69,70]. Amuvatinib, a multi-targeted tyrosine kinase inhibitor, acts on KIT, PDGFRA, MET, and RET, positioning it as a promising candidate for repurposing [71]. Regorafenib interacts with KIT, PDGFRA, RET, EPHA2, and FGFR family members connected to PIK3CA and CLDN1, providing a mechanistic basis for its application in HNC [72]. Additionally, non-kinase agents like Quercetin, identified for its pro-apoptotic and antioxidant effects, found to target ESR1, HCK, and STAT3, linked to PIK3CA, shows potenital for HNC treatment beyond conventional kinase inhibition [68]. Within both the direct risk-gene targeting analysis and the protein interaction expanded pathway analysis, XL765 (Voxtalisib) consistently emerged as a top candidate, underscoring its potential as a promising therapeutic option [51].

When comparing these results to previous TCGA genomic investigation work by Sayans et al., the 10 relevant cancer risk genes they identified had overlap with these results on PIK3CA and SOX2 [25]. Due to the HPV stratification, GARD captures genomic alterations that are significant within each HPV specific biological context. As a result, key genes that emerge in an HPV-positive landscape may not appear significant in a non-stratified analysis, leading to differences between GARD’s results and previous non-stratified methodologies. This difference in methodology explains the minimal overlap with repurposing pipelines such as Wei et al. [19]. Though it maintained a similar pipeline structure, their work was based on transcriptomic overlap with drug-treated cell lines. Combined with the absence of HPV-based stratification in their analysis, it is therefore unsurprising that the resulting drug candidates showed no overlap with the HPV-positive candidates identified in our study. Overall, HPV-positive tumors identified key risk genes that were expanded to include key signaling and targetable pathways, supporting the identification of novel therapeutics. Many were known or in clinical trials, but those that were not held potential as repurposing candidates. These findings highlight the potential for rapid translation of repurposed drugs into clinical applications for HPV-positive HNC.

### 3.3. HPV-Negative Investigation

Similar to the HPV-positive analysis, CNV and SOM analysis identified the HNC-associated genes. Table 3 shows that HPV-negative CNV amplification analysis identified 446 high-risk genes, of which 25 were available as drug targets, and led to 2945 immediate neighbors, totaling 886 targetable genes, resulting in 90 significant medications. CNV deletion analysis yielded 318 high-risk genes, of which one was available as a drug target, and led to 460 immediate neighbors totaling 127 targetable genes, resulting in 30 significant medications. Somatic mutation analysis revealed 166 high-risk genes, of which 32 were available as drug targets, and led to 2253 immediate neighbors totaling 913 targetable genes, resulting in 42 significant medications. This led to a total of 110 medications to be repurposed that targeted identified risk genes or immediate protein neighbors. Of these 110 medications, 20 were identified by directly targeting risk genes, and 104 were identified by indirectly targeting protein neighbors of risk genes. An overlap was found across 14 medications, including: 3-isobutyl-1-methyl-7h-xanthine, Acetylsalicylic Acid, Biotin, Bisindolylmaleimide i, Caffeine, Dasatinib, Ethanol, Fludiazepam, Fostamatinib, Glutamic acid, NADH, Phenethyl Isothiocyanate, Regorafenib, and Wortmannin.

Through the literature analysis, this was further refined. A total of 20 genes (Appendix A Table A4) were validated among the 224 unique risk genes found to be targetable or connected to targetable genes through PPI. Among these genes, somatic mutations identified TP53, PDE3A, MAGEC1, EPHA2, COL1A2, HRAS, REG1A, LRP1B, RAC1, CDKN2A, and PIK3CA. Amplifications derived CLDN1, DVL3, EIF4G1, PDCD10, PRKCI, RFC4, SOX2, and PIK3CA (identified through two methods). Deletions were identified in CDKN2B and CDKN2A (identified through two methods). This allowed the 110 medications identified to be reduced to 62 medications, of which 7 were found through directly targeting risk genes (Appendix A Table A5) and 60 were found through indirect targeting of validated protein neighbors of key risk genes (Appendix A Table A6). There are five drugs identified from both procedures. These were Acetylsalicylic acid, Bisindolymalemide I, Dasatinib, Fostamatinib, and Regorafenib.

The medications directly targeting key risk genes (direct targets) were associated with the validated genes, EPHA2, PIK3CA, PRKCI, and TP53. Drugs directly targeting risk genes are shown in Figure 3b, with full details provided in Appendix A Table A5. The medications directly targeting risk genes included some overlap with HPV+ direct risk gene targeting candidates such as Wortmannin and XL765, but included further candidates such as Fostamatinib, Acetylsalicylic acid, Bisindolylmaleimide I, Dasatinib, and Regorafenib. Although Wortmannin shows a significant relationship to the risk genes, it remains limited by poor selectivity and toxicity [52]. XL765’s appearance in both cohorts shows a strong relationship to HNC-related genes such as PIK3CA and the potential for repurposing [50,73]. Fostamatinib remains a strong candidate, as it is broadly applicable to many genetic targets related to HNC, and is currently in trials for HNC [63]. Acetylsalicylic acid, commonly known as Aspirin, is a pain reducer with anti-inflammatory properties. With limited information regarding HNC, initial findings prove promising, and it has shown effectiveness in other types of cancers [74]. Bisindolylmaleimide I is a compound utilized for protein kinase inhibition. It is not currently used clinically, but warrants further investigation due to its interaction with PRKCI, but due to its lack of use as a medication, remains as a lower-priority repurposing candidate [75]. Dasatinib is a tyrosine kinase inhibitor used in the treatment of leukemia, and has reached phase II clinical trials for HNC [76]. Finally, Regorafenib appeared again and shows strong inhibition of EPHA2 [72].

**Figure 5:**
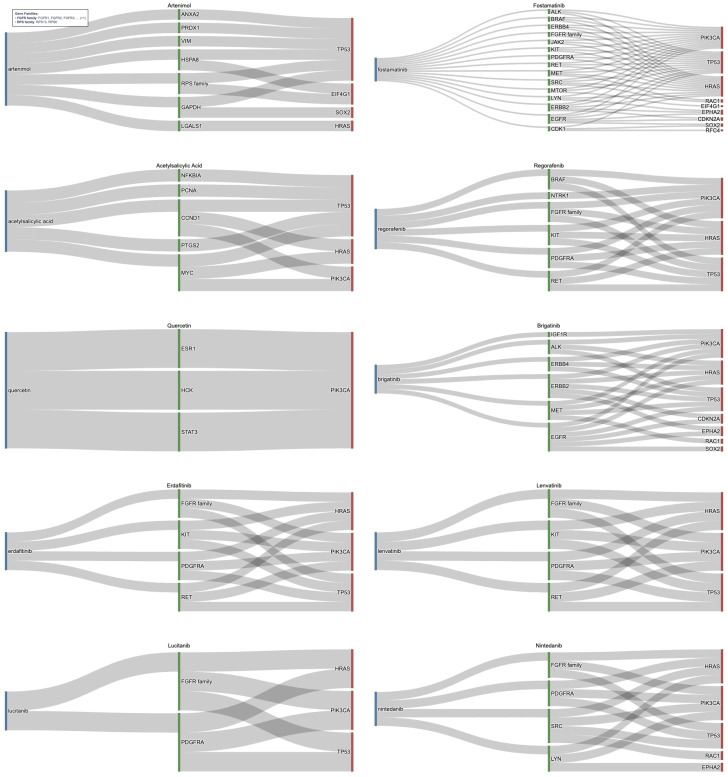
Sankey diagram for drug repurposing candidates targeting indirect genomic risk genes in HPV-negative HNC. This figure illustrates medications identified through network-based expansion of direct genomic risk genes in HPV-negative HNC using high-confidence PPI data from the STRING database. Each drug (blue) is connected to a gene target (green) that is connected to immediate neighbor risk genes (red), identified through CNV or SOM analysis. The drugs target immediate neighbor genes functionally linked to core oncogenic drivers, such as TP53, CDKN2A, and PIK3CA, representing biologically relevant therapeutic opportunities. Key candidates include multi-kinase inhibitors such as Lenvatinib and Erdafitinib, as well as drugs like Quercetin and Acetysalicylic Acid, which collectively offer promising strategies for precision oncology.

Identified risk genes were expanded through PPI networks to incorporate relevant neighbors (indirect targets). Validation of immediate neighbors and the direct risk genes were connected to reduced the 472 unique druggable immediate neighbors originally identified to a focused 77. Figure 5, which highlights the top medications identified after network expansion, shows the relationship between drugs, their gene targets, and root high-risk genes. These medications were selected from the top 50 medications ranked by FDR-adjusted significance of drug–gene associations in HPV-negative HNC (full list in Appendix A Table A6). The top medications targeted pathways stemming from connections to risk genes of TP53, PIK3CA, EIF4G1, SOX2, HRAS, RFC4, RAC1, EPHA2, and CDKN2A.

Notable candidates included multi-kinase inhibitors such as Fostamatinib, Regorafenib, Brigatinib, Erdafitinib, Lucitanib, Lenvatinib, and Nintedanib, targeting validated neighbor genes like EGFR, SRC, KIT, RET, MET, HSPA8, STAT3, PDGFRA, and the FGFR, ERBB, and RPS families. Several of these agents, Fostamatnib, Regorafenib, Brigatinib, and Nintedanib, were common to both HPV-positive and HPV-negative cohorts, reinforcing their broad mechanistic relevance. Others, such as Lenvatinib and Erdafitinib, emerged as novel candidates for HPV-negative disease. Lenvatinib, targeting the FGFR family, PDGFRA KIT, and RET, has demonstrated efficacy in HNC as part of combination regimens in clinical trials [77,78]. Erdafitinib, found to target similar genes, shows potential for repurposing in solid tumors, including HNC [79]. Lucitanib was found to target the FGFR family and PDGFRA and has shown promise in clinical trials for HNC [80]. The candidates of Fostamatnib, Regorafenib, Brigatinib, and Nintedanib were found again between the HPV+ and HPV- cohorts and shown to interact with similar key genes, such as the FGFR family, KIT, RET, PDGFRA, and SRC [63,69,72,81,82]. Beyond kinase inhibitors, other significant medications were identified. Artenimol, originally developed as an antimalarial, exhibits anticancer activity through immune modulation, targeting genes connected to TP53 and EIF4G1 [83,84]. Quercetin, previously highlighted in HPV-positive analysis, reappeared as a candidate due to its role in PI3K/AKT and STAT3 signaling [68]. Finally, Acetylsalicylic Acid (Aspirin) was found to target pathways connected to TP53, HRAS, and PIK3CA, reinforcing its potential anticancer activity and candidacy for repurposing in HNC [74]. Candidates that appeared in both avenues of analysis (direct and indirect gene targeting) included Acetylsalicylic acid, Bisindolymalemide I, Dasatinib, Fostamatinib, and Regorafenib. Many of these were strong candidates to be taken forward for analysis or are already in trials or investigations for HNC [63,72,74].

When compared with the results from the TCGA genomic study performed by Sayans et al., among their 10 key resulting genes, an overlap of TP53, CDKN2A, CDKN2B, PIK3CA, and SOX2 was observed [25]. This highlighted the ability of the pipeline to identify key genomic alterations within TCGA data. Studies, such as that published by Wei et al., show a TCGA-genomic-based cancer repurposing pipeline for HNC [19]. Though it was based around a similar pipeline, including a step for PPI expansion, this work was based on transcriptomic similarity between HNC patients in TCGA and the drug-treated cell lines. Therefore, it is not surprising that we find only one of the 22 repurposed candidate drugs for HNC identified in our drug candidates. Resveratrol, though not among the top candidates, was present among the top 50 HPV-negative indirectly repurposed drug candidates. Through the identified risk genes, alongside PPI-expanded gene pathways, key therapeutic targets were identified within the HPV-negative tumors. This led to the identification of known, or under investigation, medications alongside possible novel or exploratory repurposing candidates.

## 4. Discussion

With the expansion of available databases to utilize, drug repurposing has become a more popular and feasible way of discovering novel treatments for cancers and diseases [5]. This study introduces a genomics-driven, network-informed pipeline, GARD (Genomic Alteration-based Repurposing for Drugs), designed to systematically identify repurposing candidates for head and neck cancer (HNC). By integrating multi-omics data from HPV-stratified TCGA cohorts with high-confidence PPI networks and literature-based validation, GARD captures subtype-specific molecular signatures and expands them into biologically relevant networks. This multi-layered approach enables the identification of both direct genomic drivers and compensatory signaling hubs, which are then mapped to curated drug–gene associations from DrugBank, providing a robust framework for precision drug repurposing in HNC.

The analysis of the HPV-positive HNC cohort revealed a distinct molecular profile characterized by PIK3CA, SOX2, RFC4, CLDN1, and TLR7. Many were known cancer- or HNC-associated genes, but the incorporation of genes such as TLR7 presented underexplored targeting avenues [56,57,73,85,86,87,88]. These genes were expanded through PPI networks to further identify biologically relevant neighbors, and potential new therapeutic targets. These expanded genes included critical signaling mediators and pathway regulators (e.g., EGFR, KIT, JAK2/3, MET, RET, EPHA2, FGFR family, PDGFRA, and STAT3), which play essential roles in oncogenesis and therapeutic resistance [27,82,89,90,91]. Literature validation reinforced the mechanistic relevance of these targets, highlighting opportunities for precision drug repurposing in HPV-positive HNC. These results highlighted known pathways while uncovering novel targets, creating opportunities to identify repurposing candidates by either leveraging newly discovered gene targets or repositioning medications that act on established targets. The pipeline demonstrated its effectiveness by identifying drugs already in clinical trials for HNC, such as Buparlisib, Copanlisisb, Afatinib, Cabozantinib, Nintedanib, and Fostamatinib [45,48,59,62,81]. At the same time, it revealed promising new candidates, including XL765(Voxtalisib), Brigatinib, Amuvatinib, Deuruxolitinib, Capivasertib, and Regorafenib, which interact with critical signaling networks and represent opportunities for innovative repurposing strategies [50,67,69,71,72]. Beyond kinase inhibitors, Golotimod emerged as a unique candidate for immune modulation through TLR7 targeting [86,92]. Quercetin was also identified as a non-kinase agent with antioxidant and pro-apoptotic properties, offering unique potential to modulate PI3K/AKT and inflammatory pathways [68].

In HPV-negative tumors, alterations were dominated by cell-cycle regulators such as TP53 and CDKN2A/B, along with oncogenic drivers including PIK3CA, PRKCI, stem cell regulators like SOX2, tight-junction and EMT regulators such as CLDN1, and receptor tyrosine kinase nodes such as EPHA2 [14,56,57,73,89,93,94]. These genes were expanded through high-confidence PPI networks, revealing additional biologically relevant neighbors involved in critical pathways such as ERBB2/4, RET, KIT, EGFR, PDGFRA, MET, SRC, STAT3, FGFR family, and RPS family [27,82,90,91,95]. This network expansion broadened the therapeutic landscape by incorporating both known drivers and novel targeting opportunities not traditionally associated with HNC, such as EIF4G1. Literature-based validation reinforced the mechanistic relevance of these targets, highlighting opportunities for precision drug repurposing in HPV-negative disease. The pipeline confirmed its predictive strength by identifying drugs already in clinical trials for HNC, including Fostamatinib, Dasatinib, Lucitanib, and Lenvatinib [63,76,77,78,80]. At the same time, it uncovered novel candidates such as Brigatinib, Regorafenib, Erdafitinib, Amuvatinib, Artenimol, Acetylsalicylic acid, and Quercetin, which expand therapeutic options through possible repurposing candidates [50,67,69,71,72]. Notably, Erdafitinib, an FGFR inhibitor approved for bladder cancer, and Brigatinib, an ALK/EGFR inhibitor used in lung cancer, represent strong mechanistic candidates for repurposing in HPV-negative HNC. Beyond kinase inhibitors, Quercetin emerged as an agent with antioxidant and pro-apoptotic properties, offering unique potential to modulate PI3K/AKT and inflammatory pathways [68]. Similarly, Artenimol, originally developed as an antimalarial, demonstrated anticancer activity through oxidative stress and immune modulation, while Acetylsalicylic Acid (Aspirin) offers a low-cost anti-inflammatory strategy with emerging evidence of anticancer benefits [74,83,84].

By integrating direct genomic targets with network-based expansion, the GARD pipeline broadens the identified genes beyond known drivers, incorporating biologically relevant neighbors. The inclusion of literature-based validation further strengthens confidence in these identified targets, reducing false positives and prioritizing high-confidence targets. Identifying these gene targets and protein neighbors associated with HNC provides insight into the disease, enabling identification of drugs that can modulate these pathways, providing evidence for making them strong candidates for repurposing. This multi-layered approach allowed for the identification of known medications alongside novel candidates that have clear safety profiles and mechanistic pathways, improving the potential for translation into clinical practice. Importantly, the stratification of cohorts by HPV status proved essential for revealing distinct molecular vulnerabilities. HPV-positive and HPV-negative tumors exhibit fundamentally different drivers, which influence treatment response. By capturing these subtype-specific signatures, the GARD pipeline enables precision drug repurposing tailored to the unique biology of each subgroup, moving beyond one-size-fits-all approaches. This positions the pipeline as an advancement over previous genomic-based repurposing pipelines by filling the gaps of thorough genomic analysis of HPV stratified cohorts, inclusion of expanded relevant biological pathways, and comprehensive validation efforts. Overall, the integration of genomics data, network-based expansion, and literature validation through the GARD pipeline provides a robust and scalable framework for systematic drug repurposing in HNC and broader application across other malignancies.

This study’s reliance on TCGA data introduces limitations related to cohort size and population diversity, as such expansion through new databases or larger cohorts could provide expanded results. The reliance on HPV stratification through RNA-seq alignment carries the limitation of potential false identifications. The literature-based validation methods hold limitations to identify associations between genes and HNC, requiring manual review. The use of emerging LLM models would aid in improving the validation of causal relationships. These limitations lead the identified repurposing candidates to require further investigation. Currently, the repurposing pipeline is focused on one publicly available genomic dataset for identifying candidates and could benefit from expansion through transcriptomic or proteomic data, and other computational methods such as through real-world and EHR data [5,96]. Furthermore, while the computational predictions presented here are promising, experimental validation remains essential. Future work should focus on further validating data sources and translating these predictions into preclinical models, such as through HPV-stratified cell lines and adaptive clinical trial designs, to confirm efficacy and their use in clinical practice as viable therapeutic options. The identified candidates were a focused set of drug repurposing candidates that serve as a hypothesis to be taken through further computational, experimental and clinical evaluation. Through these methods, the identified candidates can be evaluated and validated further to translate the medication to clinical application in HNC.

## 5. Conclusions

In conclusion, the GARD pipeline identified 90 candidate medications for HPV+ HNC and 62 medications for HPV- HNC. Many medications were in initial trials for HNC and showed the viability of the pipeline for the identification of repurposing candidates. Further underexplored medications have the potential to serve as repurposing candidates in the field of HNC. The GARD pipeline demonstrates the potential for genomic-based drug repurposing and shows its potential in identifying novel treatments in HNC or further diseases. 

## Figures and Tables

**Table 1:** Input structure for LLM-based literature parsing. This table shows the standardized input format provided to Google GEMMA for parsing PubMed abstracts. Each record includes the PMID, article title, publication year, and abstract text, ensuring structured and interpretable data.

PMID	Title	Year	Abstract
Pubmed ID associated with the paper that was used	Title of the paper	Year of Publication	Abstract of the paper covering an overview of the paper and its associated results

**Table 2:** Example output structure from LLM-based literature parsing. This table illustrates the standardized format used to present results from Google GEMMA’s parsing of PubMed abstracts. Each entry includes the identified gene target, the supporting sentence from the abstract, the source publication (PMID), and associated drugs interacting with the gene. This structured approach facilitates efficient manual validation and downstream integration into drug repurposing analyses.

PMID	Title	Year	Abstract	Targets	Sentence Where Found
Pubmed ID associated with the paper that was used	Title of the paper	Year of Publication	Abstract of the paper covering an overview of the paper and its associated results	Targets identified as relevant targets for HNC through LLM analysis of the abstract	Key evidence in the abstract where the targets were found to be associated with HNC

**Table 3:** Summary of genomic and pharmacological analysis across HPV-stratified HNC cohorts. This table presents the number of high-risk genes identified through copy number amplification, deletion, and somatic mutation analyses in both HPV-positive and HPV-negative subgroups. It includes counts of direct drug targets, immediate neighbor genes identified via STRING PPI networks, drug-annotated genes from DrugBank, and the final number of statistically significant medications identified through enrichment-based filtering. These results provide a comparative overview of the molecular landscape and therapeutic potential across stratified HNC populations.

Cohort Type	High-Risk Genes	Risk Genes Available as Drug Targets	Immediate Neighbors	Drug-Annotated Genes (Neighbors + Risk Genes)	Significant Medications
HPV-Negative CNV Amplification	446	25	2945	886	87
HPV-Negative CNV Deletion	318	1	460	127	31
HPV-Negative Somatic Mutation	166	32	2253	913	46
HPV-Positive CNV Amplification	169	8	1874	627	143
HPV-Positive CNV Deletion	274	20	1518	458	42
HPV-Positive Somatic Mutation	6	1	338	135	90

## Data Availability

All data is available publicly. TCGA: access to the data is controlled as specified by The Cancer Genome Atlas project; open-access data from TCGA was utilized for this study. It is available at https://www.cancer.gov/ccg/research/genome-sequencing/tcga. https://string-db.org (accessed on 13 November 2025). STRING: STRING is a public database for protein–protein interactions available at https://string-db.org. https://go.drugbank.com (Version 12 accessed on 29 July 2025). DrugBank: drug–gene interaction data was taken from DrugBank; use of their API is through registered access. All information is available at https://go.drugbank.com. https://pubmed.ncbi.nlm.nih.gov (Version 6.0 accessed on 31 July 2025) PubMed: holds a public repository of the peer-reviewed literature. Use of API calling and programmatic scraping of data requires registration. Access is available at https://pubmed.ncbi.nlm.nih.gov (accessed on 6 August 2025).

## Abbreviations

The following abbreviations are used in this manuscript:

HNC	Head and neck cancer
TCGA	The Cancer Genome Atlas
HPV	Human Papillomavirus
PPI	Protein–Protein Interaction
FDR	False Discovery Rate
GARD	Genomic Alteration-based Repurposing for Drugs

## Appendix A

This appendix provides a detailed extension of the main analysis by presenting comprehensive tabular outputs from the drug repurposing pipeline for HNC. It includes three key components for each HPV stratified cohort: (1) tables of top genes identified through copy number variation (CNV) and somatic mutation analyses, stratified by HPV status, which highlight statistically significant drivers of disease biology validated through binomial and empirical testing; (2) tables of direct drug–gene associations, listing medications that target these high-confidence genes, supported by the literature evidence and prioritized using empirical FDR-adjusted *p*-values; and (3) tables of indirect drug–gene associations derived from network-based expansion using high-confidence STRING PPI data, showcasing the top 50 medications ranked by empirical significance. Together, these resources provide transparency into the computational workflow, illustrate the molecular distinctions between HPV-positive and HPV-negative cohorts, and support the identification of both established and novel therapeutic candidates for precision oncology in HNC.

For the code utilized in this analysis pipeline and the production of all figures and results, please refer to github link: https://github.com/pvtanike/Genomic-Landscape-Based-Drug-Repurposing.git (accessed on 17 February 2026).

**Table A1:** **Top genes identified in HPV-positive HNC cohort**, with columns for gene name, mutation type (MUT TYPE), q-value derived from binomial test, and empirical q-value derived from permutation (CNV) or multinomial (SOM) null distributions. These genes represent statistically significant drivers of disease progression, identified through CNV and somatic mutation analyses with FDR adjustment. Their inclusion highlights core oncogenic and immune-related alterations, such as amplifications in PIK3CA and deletions in TLR7, that underpin the distinct molecular landscape of HPV-positive tumors. All listed gene targets were further validated through large-scale literature mining, ensuring strong evidence of their biological relevance in HNC. These findings informed downstream drug–gene mapping and network-based expansion, ultimately guiding the identification of precision drug repurposing candidates.

Gene Name	Mutation Type	FDR-Adjusted *p*-Value	FDR-Adjusted Empirical *p*-Value	PMID	Number of Articles
CLDN1	AMPLIFICATION	6.591105 × $10^{-53}$	0.011613123	15170668, 17091452	2.0
PIK3CA	AMPLIFICATION, SOMATIC	4.9013845 × $10^{-54}$, 4.6307549 × $10^{-18}$	0.011613123, 0.0	11358835, 11836556, 11959846, 14581353, 155436…	17.0
RFC4	AMPLIFICATION	4.9013845 × $10^{-54}$	0.011613123	16467079	1.0
SOX2	AMPLIFICATION	1.292043 × $10^{-55}$	0.011613123	15942670	1.0
TLR7	DELETION	3.078243 × $10^{-8}$	0.0254037	17201162	1.0

**Table A2:** **Direct drug–gene associations for the HPV-positive HNC cohort are summarized in this table**. Columns include drug name, literature-validated gene targets, mutation type (MUT TYPE), supporting PMID references, number of articles, empirical FDR-adjusted *p*-values, and toxicity. These medications directly target high-confidence genomic alterations, such as amplifications in *PIK3CA* and deletions in *TLR7*, that define the molecular landscape of HPV-positive tumors. To ensure biological relevance, all risk genes were validated through a literature-based pipeline, and only candidates targeting genes with literature-validated associations to HNC were retained. This validation step strengthens mechanistic confidence, while empirical FDR adjustment ensures statistical robustness. These direct associations informed the prioritization of top candidates, including PI3K inhibitors (Buparlisib, Copanlisib) and immune modulators (Golotimod), which emerged as promising repurposing opportunities for precision therapy in HPV-positive HNC.

DRUG	Drug Empirical FDR Adjusted *p*-Value	Mutation Type	Literature-Validated Gene Targets	Article Count	PMIDs	Drug–Gene Interaction	Toxicity
Wortmannin	0.003022	AMPLIFICATION, SOMATIC	PIK3CA	17	14581353, 11836556, 11959846, 11358835, 16807070, …	PIK3CA: UNKNOWN	None
Golotimod	0.007807	DELETION	TLR7	1	17201162	TLR7: UNKNOWN	No adverse effects were observed in any patients participating in clinical trials.
XL765	0.010893	AMPLIFICATION, SOMATIC	PIK3CA	17	14581353, 11836556, 11959846, 11358835, 16807070, …	PIK3CA: UNKNOWN	None
Buparlisib	0.038252	AMPLIFICATION, SOMATIC	PIK3CA	17	14581353, 11836556, 11959846, 11358835, 16807070, …	PIK3CA: inhibitor	None
CH-5132799	0.038252	AMPLIFICATION, SOMATIC	PIK3CA	17	14581353, 11836556, 11959846, 11358835, 16807070, …	PIK3CA: inhibitor	None
Copanlisib	0.038252	AMPLIFICATION, SOMATIC	PIK3CA	17	14581353, 11836556, 11959846, 11358835, 16807070, …	PIK3CA: inhibitor	The LD of copanlisib dihydrochloride in male and female rats after a single intravenous dose administration was >23.0 mg/kg, which corresponds to 20.0 mg/kg copanlisib. [L48350] There is no information on copanlisib overdose.

**Table A3:** **Top 50 indirect drug–gene associations for HPV-positive HNC cohort, ranked by empirical FDR-adjusted *p*-values.** Columns include drug name, literature-validated gene targets, associated connected risk gene, mutation type (MUT TYPE), supporting PMID references, number of supporting articles, empirical FDR-adjusted *p*-values, and toxicity. These medications target immediate network neighbors of high-confidence risk genes identified through CNV and somatic mutation analyses, leveraging STRING PPI data to capture pathway-level relevance. All gene targets and their network neighbors were validated through a large-scale literature mining pipeline to ensure biological significance, and only candidates with literature-supported associations to HNC were retained. This network-based expansion broadens the therapeutic landscape beyond direct genomic alterations, uncovering multi-kinase inhibitors (e.g., Brigatinib, Capivasertib) and novel agents (Quercetin) with strong mechanistic ties to HPV-positive HNC biology.

DRUG	Drug Empirical FDR	Literature-Validated Gene Targets	Literature-Validated Risk Genes	Risk Gene Mutation Type	Gene Target Article Count	Gene Target PMIDs	Risk Gene Article Count	Risk Gene PMIDs	Drug–Gene Interaction	Toxicity
xl820	0.001703	KIT, PDGFRA	PIK3CA	AMPLIFICATION, SOMATIC	9	17253141, 16757203, 17317803, 17935283, 16630292, …	17	17990317, 11836556, 11358835, 18376308, 17549376, …	KIT: UNKNOWN; PDGFRA: UNKNOWN	None
xl999	0.001703	FGFR1, FGFR3, RET	PIK3CA	AMPLIFICATION, SOMATIC	55	11370534, 11216646, 12743496, 12670889, 11706744, …	17	17990317, 11836556, 11358835, 18376308, 17549376, …	FGFR1: UNKNOWN; FGFR3: UNKNOWN; RET: UNKNOWN	None
2-tert-butyl-9-fluoro-1,6-dihydrobenzo [h]imidazo [4,5-f] isoquinolin-7-one	0.001703	JAK2, JAK3	PIK3CA	AMPLIFICATION, SOMATIC	2	18204781, 15947106	17	17990317, 11836556, 11358835, 18376308, 17549376, …	JAK2: inhibitor; JAK3: inhibitor	None
su-11652	0.001703	PDGFRA	PIK3CA	AMPLIFICATION, SOMATIC	2	14499691, 17513510	17	17990317, 11836556, 11358835, 18376308, 17549376, …	PDGFRA: inhibitor	None
xl765	0.001703	MTOR	PIK3CA	AMPLIFICATION, SOMATIC	8	15379322, 17047074, 16927414, 15833854, 18089792, …	17	17990317, 11836556, 11358835, 18376308, 17549376, …	MTOR: UNKNOWN	None
entrectinib	0.001703	ALK, JAK2, NTRK1, NTRK3	PIK3CA	AMPLIFICATION, SOMATIC	10	15947106, 11216646, 17414097, 16796648, 11279608, …	17	17990317, 11836556, 11358835, 18376308, 17549376, …	ALK: inhibitor; JAK2: inhibitor; NTRK1: inhibitor; NTRK3: inhibitor	None
inositol 1,3,4,5-tetrakisphosphate	0.001703	AKT1	PIK3CA	AMPLIFICATION, SOMATIC	6	17974918, 12532415, 15896313, 16778075, 16002527, …	17	17990317, 11836556, 11358835, 18376308, 17549376, …	AKT1: inhibitor	None
bosutinib	0.001703	HCK, LYN, SRC	PIK3CA	AMPLIFICATION, SOMATIC	3	17252232, 16224162, 17961551	17	17990317, 11836556, 11358835, 18376308, 17549376, …	HCK: inhibitor; LYN: inhibitor; SRC: inhibitor	In a rat fertility and early embryonic development study, bosutinib was administered orally to female rats for approximately 3 to 6 weeks, depending on the day of mating (2 weeks prior to cohabitation with untreated breeder males until gestation…
brigatinib	0.002230	ALK, EGFR, ERBB2, ERBB4, IGF1R, MET	PIK3CA	AMPLIFICATION, SOMATIC	725	17949783, 16890793, 18045070, 12599217, 11939730, …	34	17990317, 11836556, 11358835, 18376308, 17549376, …	ALK: inhibitor; EGFR: inhibitor; ERBB2: inhibitor; ERBB4: inhibitor; IGF1R: inhibitor; MET: inhibitor	Treatment with brigatinib generated not mutagenic chromosomal damage. Testicular toxicity, reported as lower weight of seminal vesicles and prostate gland, testicular tubular degeneration and lower weight as well as reduced size of testes with ev…
erdafitinib	0.002230	FGFR1, FGFR2, FGFR3, FGFR4, KIT, PDGFRA, RET	PIK3CA	AMPLIFICATION, SOMATIC	145	11370534, 16757203, 17935283, 11216646, 12743496, …	34	17990317, 11836556, 11358835, 18376308, 17549376, …	FGFR1: inhibitor; FGFR2: inhibitor; FGFR3: inhibitor; FGFR4: inhibitor; KIT: substrate; PDGFRA: substrate; RET: substrate	Based on the mechanism of action and findings in animal reproduction studies, erdafitinib can cause fetal harm when administered to a pregnant woman. There are no available data on erdafitinib use in pregnant women to inform a drug-associated ris…
lenvatinib	0.002230	FGFR1, FGFR2, FGFR3, FGFR4, KIT, PDGFRA, RET	PIK3CA	AMPLIFICATION, SOMATIC	149	11370534, 16757203, 17935283, 11216646, 12743496, …	34	17990317, 11836556, 11358835, 18376308, 17549376, …	FGFR1: inhibitor; FGFR2: inhibitor; FGFR3: inhibitor; FGFR4: inhibitor; KIT: inhibitor; PDGFRA: inhibitor; RET: inhibitor	The most common adverse events that occurred in lenvatinib recipients were hypertension (67.8 vs. 9.2 % in the placebo group), diarrhea (59.4 vs. 8.4 %), fatigue or asthenia (59.0 vs. 27.5 %), decreased appetite (50.2 vs. 11.5 %), decreased bodyw…
linifanib	0.002230	KIT	PIK3CA	AMPLIFICATION, SOMATIC	21	17253141, 16757203, 17317803, 17935283, 16630292, …	34	17990317, 11836556, 11358835, 18376308, 17549376, …	KIT: UNKNOWN	None
lucitanib	0.002230	FGFR1, FGFR2, FGFR3, PDGFRA	PIK3CA	AMPLIFICATION, SOMATIC	31	11605053, 18059337, 17513510, 12569015, 11562460, …	34	17990317, 11836556, 11358835, 18376308, 17549376, …	FGFR1: inhibitor; FGFR2: inhibitor; FGFR3: inhibitor; PDGFRA: inhibitor	None
nintedanib	0.002230	FGFR1, FGFR2, FGFR3, LYN, PDGFRA, SRC	PIK3CA	AMPLIFICATION, SOMATIC	35	11605053, 17961551, 17252232, 18059337, 17513510, …	34	17990317, 11836556, 11358835, 18376308, 17549376, …	FGFR1: inhibitor; FGFR2: inhibitor; FGFR3: inhibitor; LYN: inhibitor; PDGFRA: inhibitor; SRC: inhibitor	Experience with nintedanib overdose is limited, but patients who inadvertently received higher-than-intended doses during initial trials experienced adverse effects consistent with the known safety profile of nintedanib, for example elevated live…
pd-166326	0.002230	EGFR, FGFR1, KIT, PDGFRA, SRC	PIK3CA	AMPLIFICATION, SOMATIC	629	17949783, 12826036, 15736426, 14751528, 18268492, …	34	17990317, 11836556, 11358835, 18376308, 17549376, …	EGFR: inhibitor; FGFR1: inhibitor; KIT: inhibitor; PDGFRA: inhibitor; SRC: inhibitor	None
sorafenib	0.002230	BRAF, EGFR, FGFR1, KIT, RET	PIK3CA	AMPLIFICATION, SOMATIC	1093	17949783, 16890793, 18045070, 12599217, 11939730, …	34	17990317, 11836556, 11358835, 18376308, 17549376, …	BRAF: inhibitor; EGFR: inhibitor; FGFR1: inhibitor; KIT: antagonist; RET: inhibitor	The oral lowest published toxic dose (Toxic Dose Low, TDLo) is 2.84 mg/kg/21D (intermittent). [L44582] The oral LD50 of sorafenib tosylate in rats is >2000 mg/kg. [L44607] The adverse reactions observed at 800 mg sorafenib twice d…
sunitinib	0.002230	KIT, MET, PDGFRA	PIK3CA	AMPLIFICATION, SOMATIC	60	16757203, 17075124, 17935283, 17513510, 17684930, …	34	17990317, 11836556, 11358835, 18376308, 17549376, …	KIT: inhibitor; MET: inhibitor; PDGFRA: inhibitor	The maximally tolerated dose for rat, mouse, and dog when given orally is greater than 500 mg/kg. The maximally tolerated dose of a non-human primate is greater 1200 mg/kg.
pralsetinib	0.002230	FGFR1, FGFR2, JAK2, NTRK1, NTRK3, RET	PIK3CA	AMPLIFICATION, SOMATIC	118	11370534, 11216646, 12743496, 12670889, 11706744, …	34	17990317, 11836556, 11358835, 18376308, 17549376, …	FGFR1: inhibitor; FGFR2: inhibitor; JAK2: inhibitor; NTRK1: inhibitor; NTRK3: inhibitor; RET: inhibitor	Pralsetinib administered to rats at 20 mg/kg (roughly 2.5–3.6 times the recommended human exposure) resulted in resorption of litters in pregnant female mice in 92% of pregnancies (82% complete resorption); resorption occurred at doses as low as…
tivozanib	0.002230	FGFR1, KIT, MET, PDGFRA	PIK3CA	AMPLIFICATION, SOMATIC	70	16757203, 17075124, 17935283, 17513510, 17684930, …	34	17990317, 11836556, 11358835, 18376308, 17549376, …	FGFR1: inhibitor; KIT: inhibitor; MET: inhibitor; PDGFRA: inhibitor	LD50 information for tivozanib is not readily available in the literature, however the European Medicines Agency (EMA) assessment report indicates the oral maximum tolerated dose (MTD) in mice was 524 mg/kg; doses ≥750 mg/kg led to severe and le…
pp-121	0.002230	EGFR, HCK, MTOR, PDGFRA, SRC	PIK3CA	AMPLIFICATION, SOMATIC	616	17949783, 16890793, 18045070, 12599217, 11939730, …	34	17990317, 11836556, 11358835, 18376308, 17549376, …	EGFR: inhibitor; HCK: inhibitor; MTOR: inhibitor; PDGFRA: inhibitor; SRC: inhibitor	None
ponatinib	0.002230	FGFR1, FGFR2, FGFR3, FGFR4, KIT, LYN, PDGFRA, RET, SRC	PIK3CA	AMPLIFICATION, SOMATIC	153	11370534, 16757203, 17935283, 11216646, 12743496, …	34	17990317, 11836556, 11358835, 18376308, 17549376, …	FGFR1: inhibitor; FGFR2: inhibitor; FGFR3: inhibitor; FGFR4: inhibitor; KIT: inhibitor; LYN: inhibitor; PDGFRA: inhibitor; RET: inhibitor; SRC: inhibitor	The most common non-hematologic adverse reactions (≥20%) were hypertension, rash, abdominal pain, fatigue, headache, dry skin, constipation, arthralgia, nausea, and pyrexia. Hematologic adverse reactions included thrombocytopenia, anemia, neutro…
regorafenib	0.002230	BRAF, EPHA2, FGFR1, FGFR2, KIT, NTRK1, PDGFRA, RET	CLDN1, PIK3CA	AMPLIFICATION, SOMATIC	228	11370534, 17525478, 17471236, 17638058, 16551863, …	36	16815198, 11959846, 18447972, 18376308, 16676365, …	BRAF: inhibitor; EPHA2: inhibitor; FGFR1: inhibitor; FGFR2: inhibitor; KIT: inhibitor; NTRK1: inhibitor; PDGFRA: inhibitor; RET: inhibitor	The most common adverse reactions (≥20%) are asthenia/fatigue, HFSR, diarrhea, decreased appetite/food intake, hypertension, mucositis, dysphonia, and infection, pain (not otherwise specified), decreased weight, gastrointestinal and abdominal pai…
zanubrutinib	0.002230	EGFR, ERBB2, ERBB4, JAK2, JAK3	PIK3CA	AMPLIFICATION, SOMATIC	654	17949783, 16890793, 18045070, 12599217, 11939730, …	34	17990317, 11836556, 11358835, 18376308, 17549376, …	EGFR: inhibitor; ERBB2: inhibitor; ERBB4: inhibitor; JAK2: inhibitor; JAK3: inhibitor	There is limited data on zanubrutinib overdose.
1-tert-butyl-3-(4-chloro-phenyl)-1h-pyrazolo[3,4-d]pyrimidin-4-ylamine	0.003022	LYN, SRC	PIK3CA	AMPLIFICATION, SOMATIC	2	17252232, 17961551	17	17990317, 11836556, 11358835, 18376308, 17549376, …	LYN: UNKNOWN; SRC: UNKNOWN	None
altiratinib	0.003022	MET, NTRK1, NTRK3	PIK3CA	AMPLIFICATION, SOMATIC	15	11752453, 17075124, 11216646, 15319714, 11279608, …	17	17990317, 11836556, 11358835, 18376308, 17549376, …	MET: inhibitor; NTRK1: inhibitor; NTRK3: inhibitor	None
ruxolitinib	0.003022	JAK2, JAK3	PIK3CA	AMPLIFICATION, SOMATIC	2	18204781, 15947106	17	17990317, 11836556, 11358835, 18376308, 17549376, …	JAK2: inhibitor; JAK3: inhibitor	The oral LD50 was 250 mg/kg. [L31953] Single doses of ruxolitinib up to 200 mg were tolerated well. Higher doses than recommended repeat doses are associated with myelosuppression, including leukopenia, anemia, and thrombocytopen…
sf1126	0.003022	MTOR	PIK3CA	AMPLIFICATION, SOMATIC	8	15379322, 17047074, 16927414, 15833854, 18089792, …	17	17990317, 11836556, 11358835, 18376308, 17549376, …	MTOR: UNKNOWN	None
bisindolylmaleimide i	0.003022	AKT1, PRKCI	PIK3CA, SOX2	AMPLIFICATION, SOMATIC	8	17974918, 17990328, 12532415, 15896313, 16778075, …	36	16815198, 11959846, 18447972, 18376308, 16676365, …	AKT1: inhibitor; PRKCI: UNKNOWN	None
famitinib	0.003747	KIT, PDGFRA	PIK3CA	AMPLIFICATION, SOMATIC	27	17253141, 16757203, 17317803, 17935283, 16630292, …	34	17990317, 11836556, 11358835, 18376308, 17549376, …	KIT: inhibitor; PDGFRA: inhibitor	None
midostaurin	0.003747	KIT, PDGFRA	PIK3CA	AMPLIFICATION, SOMATIC	18	17253141, 16757203, 17317803, 17935283, 16630292, …	34	17990317, 11836556, 11358835, 18376308, 17549376, …	KIT: antagonist; PDGFRA: antagonist	In a fertility study involving female and male rats, there is evidence of reproductive toxicity including reduced sperm count and decline pregnancy rates when administering 0.01 to 0.1 times the recommended dose in humans. Incidences of pulmonary…
selpercatinib	0.003747	FGFR1, FGFR2, FGFR3, RET	PIK3CA	AMPLIFICATION, SOMATIC	119	11370534, 11216646, 12743496, 12670889, 11706744, …	34	17990317, 11836556, 11358835, 18376308, 17549376, …	FGFR1: inhibitor; FGFR2: inhibitor; FGFR3: inhibitor; RET: inhibitor	Toxicity information regarding selpercatinib is not readily available. Patients experiencing an overdose are at an increased risk of severe adverse effects such as hepatotoxicity, hypertension, prolonged QT interval, and hemorrhaging. Symptomatic…
pazopanib	0.003747	FGFR3, KIT, PDGFRA	PIK3CA	AMPLIFICATION, SOMATIC	36	16757203, 17253141, 17317803, 17935283, 11605053, …	34	17990317, 11836556, 11358835, 18376308, 17549376, …	FGFR3: inhibitor; KIT: inhibitor; PDGFRA: inhibitor	None
fostamatinib	0.003747	ALK, BRAF, CDK4, EGFR, ERBB2, ERBB4, FGFR1, FGFR2, FGFR3, FYN, HCK, JAK2, JAK3, KIT, LYN, MET, MTOR, NTRK1, NTRK3, PDGFRA, PRKCI, RET, SRC, SYK	PIK3CA, SOX2	AMPLIFICATION, SOMATIC	962	17949783, 16890793, 18045070, 12599217, 11939730, …	38	16815198, 11959846, 18447972, 18376308, 16676365, …	ALK: inhibitor; BRAF: inhibitor; CDK4: inhibitor; EGFR: inhibitor; ERBB2: inhibitor; ERBB4: inhibitor; FGFR1: inhibitor; FGFR2: inhibitor; FGFR3: inhibitor; FYN: inhibitor; HCK: inhibitor; JAK2: inhibitor; JAK3: inhibitor; KIT: inhibitor; LYN: in…	Neither fostamatinib or R406 were found to be carcinogenic or mutagenic [FDA Label]. Fostamatinib can cause embryo-fetal mortality or developmental abnormalities at exposures of 0.3–10 times the maximum recommended human dose. Serious adve…
osi-930	0.004258	KIT	PIK3CA	AMPLIFICATION, SOMATIC	7	17253141, 16757203, 17317803, 17935283, 16630292, …	17	17990317, 11836556, 11358835, 18376308, 17549376, …	KIT: modulator	None
phosphonotyrosine	0.004258	HCK, KIT, SRC	PIK3CA	AMPLIFICATION, SOMATIC	9	16757203, 17253141, 17317803, 17935283, 17961551, …	17	17990317, 11836556, 11358835, 18376308, 17549376, …	HCK: UNKNOWN; KIT: inhibitor; SRC: inhibitor	None
foreskin fibroblast (neonatal)	0.004258	TNF	TLR7	DELETION	6	11926139, 18197435, 12569015, 16888677, 12075207, …	1	17201162	TNF: agonist	No serious adverse events were attributed to Dermagraft. Of the 314 patients enrolled in a preclinical study, 10.4% (17/163) of the patients developed an infection while 17.9% (27/151) of the control group patients developed an ulcer infection. O…
bimiralisib	0.004996	MTOR	PIK3CA	AMPLIFICATION, SOMATIC	8	15379322, 17047074, 16927414, 15833854, 18089792, …	17	17990317, 11836556, 11358835, 18376308, 17549376, …	MTOR: inhibitor	None
bms-754807	0.004996	AKT1, IGF1R	PIK3CA	AMPLIFICATION, SOMATIC	7	17974918, 12532415, 15896313, 15221937, 16778075, …	17	17990317, 11836556, 11358835, 18376308, 17549376, …	AKT1: inhibitor; IGF1R: inhibitor	None
cabozantinib	0.004996	MET, RET	PIK3CA	AMPLIFICATION, SOMATIC	58	11370534, 17075124, 11216646, 12743496, 12670889, …	17	17990317, 11836556, 11358835, 18376308, 17549376, …	MET: antagonist; RET: antagonist	Cabozantinib carries a warning of serious gastrointestinal fistulas and perforations, and potentially fatal hemoptysis and gastrointestinal hemorrhage.
capivasertib	0.004996	AKT1, AKT2	PIK3CA	AMPLIFICATION, SOMATIC	8	17974918, 15543611, 12532415, 15896313, 16778075, …	17	17990317, 11836556, 11358835, 18376308, 17549376, …	AKT1: inhibitor; AKT2: inhibitor	Based on findings in animals and mechanism of action, capivasertib can cause fetal harm when administered to a pregnant woman. There are no available data on the use of capivasertib in pregnant women. In an animal reproduction study, oral adminis…
debio-1347	0.004996	FGFR1, FGFR2, FGFR3	PIK3CA	AMPLIFICATION, SOMATIC	10	11605053, 18059337, 12569015, 11562460, 15165306, …	17	17990317, 11836556, 11358835, 18376308, 17549376, …	FGFR1: inhibitor; FGFR2: inhibitor; FGFR3: inhibitor	None
deuruxolitinib	0.004996	JAK2	PIK3CA	AMPLIFICATION, SOMATIC	1	15947106	17	17990317, 11836556, 11358835, 18376308, 17549376, …	JAK2: inhibitor	There is no experience regarding human overdose with deuruxolitinib. There is no specific antidote for overdose with deuruxolitinib. Treatment should be symptomatic and supportive and monitor patients for signs and symptoms of adverse reactions.[…
gsk-1059615	0.004996	MTOR, PIK3C3	PIK3CA	AMPLIFICATION, SOMATIC	10	15379322, 17047074, 16927414, 15833854, 18649358, …	17	17990317, 11836556, 11358835, 18376308, 17549376, …	MTOR: inhibitor; PIK3C3: inhibitor	None
pd-168393	0.004996	EGFR, ERBB2, SRC	PIK3CA	AMPLIFICATION, SOMATIC	321	17949783, 16890793, 18045070, 12599217, 11939730, …	17	17990317, 11836556, 11358835, 18376308, 17549376, …	EGFR: inhibitor; ERBB2: inhibitor; SRC: UNKNOWN	None
ritlecitinib	0.004996	JAK3	PIK3CA	AMPLIFICATION, SOMATIC	1	18204781	17	17990317, 11836556, 11358835, 18376308, 17549376, …	JAK3: inhibitor	During clinical trials, the highest single dose of ritlecitinib was 800 mg. No specific toxicities were identified at this dose, and the adverse reactions detected were comparable to those seen at lower doses. Pharmacokinetic studies indicate tha…
semaxanib	0.005303	FGFR1, KIT, PDGFRA, RET	PIK3CA	AMPLIFICATION, SOMATIC	129	11370534, 16757203, 17935283, 11216646, 12743496, …	34	17990317, 11836556, 11358835, 18376308, 17549376, …	FGFR1: inhibitor; KIT: inhibitor; PDGFRA: inhibitor; RET: inhibitor	None
quercetin	0.005303	ESR1, HCK, STAT3	PIK3CA	AMPLIFICATION, SOMATIC	44	16158919, 17122156, 11426647, 17145757, 17961551, …	17	17990317, 11836556, 11358835, 18376308, 17549376, …	ESR1: UNKNOWN; HCK: UNKNOWN; STAT3: inhibitor	None
fedratinib	0.006083	JAK2	PIK3CA	AMPLIFICATION, SOMATIC	1	15947106	17	17990317, 11836556, 11358835, 18376308, 17549376, …	JAK2: inhibitor	Data regarding fedratinib in overdose is not readily available. [L8090] Patients given 680mg/day experienced a greater incidence and severity of adverse effects including anemia, thrombocytopenia, gastrointestinal toxicity, hepatic toxicity, and e…
m-2698	0.006083	AKT1	PIK3CA	AMPLIFICATION, SOMATIC	6	17974918, 12532415, 15896313, 16778075, 16002527, …	17	17990317, 11836556, 11358835, 18376308, 17549376, …	AKT1: inhibitor	None
amuvatinib	0.006574	KIT, MET, PDGFRA, RET	PIK3CA	AMPLIFICATION, SOMATIC	154	11370534, 16757203, 17075124, 17935283, 11216646, …	34	17990317, 11836556, 11358835, 18376308, 17549376, …	KIT: modulator; MET: UNKNOWN; PDGFRA: modulator; RET: UNKNOWN	None

**Table A4:** **Top high-risk genes identified in HPV-negative HNC cohort**. This figure highlights the most significant genes identified through integrative analysis of copy number variation (CNV) and somatic mutation profiles in HPV-negative tumors. Genes such as SOX2, PRKCI, EPHA2, PIK3CA and TP53 emerged as key oncogenic drivers, alongside pathway-level regulators like EIF4G1 and CLDN1. All listed genes demonstrated strong statistical significance (FDR-adjusted *p*-value < 1.25 ×$10^{-7}$; FDR-adjusted empirical *p*-value ≤ 0.003858), indicating recurrent amplifications or somatic mutations across the cohort. These targets underpin critical biological processes, including chromatin remodeling, signal transduction, and immune modulation, and were prioritized for downstream drug repurposing analysis.

Gene Name	Mutation Type	FDR-adjusted *p*-Value	FDR-adjusted Empirical *p*-Value	PMID	Number of Articles
TP53	SOMATIC	0.0	0.0	11390535, 11445847, 11445859, 11916556, 125898…	29.0
PDE3A	SOMATIC	1.25333691318769 × $10^{-7}$	0.0	15078486	1.0
MAGEC1	SOMATIC	1.49657026360729 × $10^{-13}$	0.0	16929165	1.0
EPHA2	SOMATIC	1.56156226502966 × $10^{-16}$	0.0	12494475, 16309192, 18030354, 18425361, 18485799	5.0
COL1A2	SOMATIC	2.01838090818069 × $10^{-7}$	0.0	18254958	1.0
HRAS	SOMATIC	2.21377392298683 × $10^{-21}$	0.0	16676365	1.0
REG1A	SOMATIC	3.56242997269542 × $10^{-13}$	0.0	12901795	1.0
LRP1B	SOMATIC	4.22883913503266 × $10^{-46}$	0.0	15172977, 16857411, 16918994	3.0
RAC1	SOMATIC	4.60603810391867 × $10^{-5}$	0.0	17234718, 17592548	2.0
BCL6	AMPLIFICATION	0.0	0.0038533077280929	11224600, 11420458, 14685876, 17429099	4.0
CLDN1	AMPLIFICATION	0.0	0.0038533077280929	15170668, 17091452	2.0
DVL3	AMPLIFICATION	0.0	0.0038533077280929	14676830, 16865240	2.0
EIF4G1	AMPLIFICATION	0.0	0.0038533077280929	14676830	1.0
PDCD10	AMPLIFICATION	0.0	0.0038533077280929	17409414	1.0
PRKCI	AMPLIFICATION	0.0	0.0038533077280929	17990328	1.0
RFC4	AMPLIFICATION	0.0	0.0038533077280929	16467079	1.0
SOX2	AMPLIFICATION	0.0	0.0038533077280929	15942670	1.0
PIK3CA	AMPLIFICATION, SOMATIC	0.0, 2.110354630735758 × $10^{-49}$	0.0038533077280929, 0.0	11358835, 11836556, 11959846, 14581353, 155436…	17.0
CDKN2B	DELETION	6.99787817318417 × $10^{-279}$	0.0204188933116782	12632081, 17673925	2.0
CDKN2A	DELETION, SOMATIC	7.356022172760123 × $10^{-290}$, 4.632181064319507 × $10^{-128}$	0.0204188933116782, 0.0	11309301, 11720478, 12459644, 12632081, 126844…	16.0

**Table A5:** **Direct drug–gene associations identified for the HPV-negative HNC cohort** are summarized in this table. Columns include drug name, literature-validated gene targets, mutation type (MUT TYPE), supporting PMID references, number of supporting articles, empirical FDR-adjusted *p*-values, and toxicity. These medications directly target high-confidence genomic alterations, such as amplifications in PRKCI, and PIK3CA, as well as somatic mutations in TP53 and EPHA2, which define the molecular landscape of HPV-negative tumors. All gene targets were validated through a large-scale literature-mining pipeline to ensure biological relevance, and only candidates with confirmed associations to HNC were retained. Literature validation strengthens mechanistic confidence, while empirical FDR adjustment ensures statistical robustness. These direct associations informed the prioritization of top candidates, including multi-kinase inhibitors (Fostamatinib, Regorafenib) and pathway modulators (Acetylsalicylic acid), which emerged as promising repurposing opportunities for precision therapy in HPV-negative HNC.

DRUG	Drug FDR-adjusted Empirical *p*-Value	Mutation Type	Literature-Validated Gene Targets	Article Count	PMIDs	Drug–Gene Interaction	Toxicity
Bisindolylmaleimide I	0.002927	AMPLIFICATION	PRKCI	1	17990328	PRKCI: UNKNOWN	None
Fostamatinib	0.002927	AMPLIFICATION, SOMATIC	EPHA2, PRKCI	6	18485799, 17990328, 12494475, 18030354, 18425361, …	EPHA2: inhibitor; PRKCI: inhibitor	Neither fostamatinib or R406 were found to be carcinogenic or mutagenic [FDA Label]. Fostamatinib can cause embryo-fetal mortality or developmental abnormalities at exposures of 0.3–10 times the maximum recommended human dose. Serious adverse effects include hypertension, neutropenia, diarrhea, and hepatotoxicity [FDA Label]
Acetylsalicylic acid	0.004258	SOMATIC	TP53	29	11445847, 11445859, 12702551, 17312781, 16455286, …	TP53: inducer	**Lethal doses** Acute oral LD50 values have been reported as over 1.0 g/kg in humans, cats, and dogs, 0.92–1.48 g/kg in albino rats, 1.19 g/kg in guinea pigs, 1.1 g/kg in mice, and 1.8 g/kg in rabbit models [FDA label]. **Acute toxicity** Salicylate toxicity is a problem that may develop with both acute and chronic salicylate exposure [A35408]. Multiple organ systems may be affected by salicylate toxicity, including the central nervous system, the pulmonary s…
Dasatinib	0.004258	SOMATIC	EPHA2	5	18485799, 12494475, 18030354, 18425361, 16309192	EPHA2: antagonist	Overdose cases with dasatinib occurred in isolated cases during clinical studies. Patients that received 280 mg of dasatinib per day for 1 week developed severe myelosuppression and bleeding. Since dasatinib is associated with severe myelosuppression, patients that ingest more than the recommended dosage should be monitored closely for myelosuppression and receive appropriate supportive treatment. Acute overdose in animals was associated with cardiotoxicity. In rodents, ventri…
Regorafenib	0.004258	SOMATIC	EPHA2	5	18485799, 12494475, 18030354, 18425361, 16309192	EPHA2: inhibitor	The most common adverse reactions (≥20%) are asthenia/fatigue, HFSR, diarrhea, decreased appetite/food intake, hypertension, mucositis, dysphonia, and infection, pain (not otherwise specified), decreased weight, gastrointestinal and abdominal pain, rash, fever, and nausea
Wortmannin	0.046275	AMPLIFICATION, SOMATIC	PIK3CA	17	14581353, 11836556, 11959846, 11358835, 16807070, …	PIK3CA: UNKNOWN	None
XL765	0.046301	AMPLIFICATION, SOMATIC	PIK3CA	17	14581353, 11836556, 11959846, 11358835, 16807070, …	PIK3CA: UNKNOWN	None

**Table A6:** **Top 50 indirect drug–gene associations for the HPV-negative HNC cohort**, ranked by empirical FDR-adjusted *p*-values. Columns include drug name, literature-validated gene targets, associated connected risk gene, mutation type (MUT TYPE), supporting PMID references, number of supporting articles, empirical FDR-adjusted *p*-values, and toxicity. These medications target immediate network neighbors of high-confidence risk genes identified through CNV and somatic mutation analyses, leveraging STRING PPI data to capture pathway-level relevance. All gene targets and their network neighbors were validated through a large-scale literature-mining pipeline to ensure biological significance, and only candidates with confirmed associations to HNC were retained. This network-based expansion broadens the therapeutic landscape beyond direct genomic alterations, uncovering multi-kinase inhibitors (e.g., Brigatinib and Regorafenib) and novel agents (Fostamatinib, Artenimol, Quercetin) with strong mechanistic ties to HPV-negative HNC biology. Literature validation and statistical prioritization reinforce the translational potential of these candidates for precision drug repurposing.

DRUG	DrugEmpirical FDR	Literature-Validated Gene Targets	Literature-Validated Risk Genes	Risk Gene Mutation Type	Gene Target Article Count	Gene Target PMIDs	Risk Gene Article Count	Risk Gene PMIDs	Drug–Gene Interaction	Toxicity
brigatinib	0.004258	ALK, EGFR, ERBB2, ERBB4, IGF1R, MET	CDKN2A, PIK3CA	AMPLIFICATION, DELETION, SOMATIC	1004	17949783, 16890793, 18045070, 12599217, 11939730, …	50	16380997, 16676365, 16849682, 17079134, 16278815, …	ALK: inhibitor; EGFR: inhibitor; ERBB2: inhibitor; ERBB4: inhibitor; IGF1R: inhibitor; MET: inhibitor	Treatment with brigatinib generated not mutagenic chromosomal damage. Testicular toxicity, reported as lower weight of seminal vesicles and prostate gland, testicular tubular degeneration and lower weight as well as reduced size of testes with ev…
erdafitinib	0.004258	FGFR1, FGFR2, FGFR3, FGFR4, KIT, PDGFRA, RET	PIK3CA	AMPLIFICATION, SOMATIC	134	11370534, 16757203, 17935283, 11216646, 12743496, …	34	17549376, 11358835, 15543611, 16380997, 18376308, …	FGFR1: inhibitor; FGFR2: inhibitor; FGFR3: inhibitor; FGFR4: inhibitor; KIT: substrate; PDGFRA: substrate; RET: substrate	Based on the mechanism of action and findings in animal reproduction studies, erdafitinib can cause fetal harm when administered to a pregnant woman. There are no available data on erdafitinib use in pregnant women to inform a drug-associated ris…
lenvatinib	0.004258	FGFR1, FGFR2, FGFR3, FGFR4, KIT, PDGFRA, RET	PIK3CA	AMPLIFICATION, SOMATIC	134	11370534, 16757203, 17935283, 11216646, 12743496, …	34	17549376, 11358835, 15543611, 16380997, 18376308, …	FGFR1: inhibitor; FGFR2: inhibitor; FGFR3: inhibitor; FGFR4: inhibitor; KIT: inhibitor; PDGFRA: inhibitor; RET: inhibitor	The most common adverse events that occurred in lenvatinib recipients were hypertension (67.8 vs. 9.2% in the placebo group), diarrhea (59.4 vs. 8.4%), fatigue or asthenia (59.0 vs. 27.5%), decreased appetite (50.2 vs. 11.5%), decreased bodyw…
lucitanib	0.004258	FGFR1, FGFR2, FGFR3, PDGFRA	PIK3CA	AMPLIFICATION, SOMATIC	24	11605053, 18059337, 17513510, 12569015, 11562460, …	34	17549376, 11358835, 15543611, 16380997, 18376308, …	FGFR1: inhibitor; FGFR2: inhibitor; FGFR3: inhibitor; PDGFRA: inhibitor	None
nintedanib	0.004258	FGFR1, FGFR2, FGFR3, LYN, PDGFRA, SRC	PIK3CA	AMPLIFICATION, SOMATIC	28	11605053, 17961551, 17252232, 18059337, 17513510, …	34	17549376, 11358835, 15543611, 16380997, 18376308, …	FGFR1: inhibitor; FGFR2: inhibitor; FGFR3: inhibitor; LYN: inhibitor; PDGFRA: inhibitor; SRC: inhibitor	Experience with nintedanib overdose is limited, but patients who inadvertently received higher-than-intended doses during initial trials experienced adverse effects consistent with the known safety profile of nintedanib, for example elevated live…
pd-166326	0.004258	EGFR, FGFR1, KIT, PDGFRA, SRC	CDKN2A, PIK3CA, TP53	AMPLIFICATION, DELETION, SOMATIC	921	17949783, 12826036, 15736426, 14751528, 18268492, …	79	16380997, 16676365, 16682429, 16849682, 12702551, …	EGFR: inhibitor; FGFR1: inhibitor; KIT: inhibitor; PDGFRA: inhibitor; SRC: inhibitor	None
sorafenib	0.004258	BRAF, EGFR, FGFR1, KIT, RET	PIK3CA	AMPLIFICATION, SOMATIC	790	17949783, 16890793, 18045070, 12599217, 11939730, …	34	17549376, 11358835, 15543611, 16380997, 18376308, …	BRAF: inhibitor; EGFR: inhibitor; FGFR1: inhibitor; KIT: antagonist; RET: inhibitor	The oral lowest published toxic dose (Toxic Dose Low, TDLo) is 2.84 mg/kg/21D (intermittent). [L44582] The oral LD50 of sorafenib in rats is >2000 mg/kg. [L44607] The adverse reactions observed at 800 mg sorafenib twice d…
sunitinib	0.004258	KIT, MET, PDGFRA	PIK3CA	AMPLIFICATION, SOMATIC	40	16757203, 17075124, 17935283, 17513510, 17684930, …	34	17549376, 11358835, 15543611, 16380997, 18376308, …	KIT: inhibitor; MET: inhibitor; PDGFRA: inhibitor	The maximally tolerated dose for rat, mouse, and dog when given orally is greater than 500 mg/kg. The maximally tolerated dose of a non-human primate is greater 1200 mg/kg.
ponatinib	0.004258	FGFR1, FGFR2, FGFR3, FGFR4, KIT, LYN, PDGFRA, RET, SRC	CDKN2A, PIK3CA	AMPLIFICATION, DELETION, SOMATIC	144	11370534, 16757203, 17935283, 11216646, 12743496, …	50	16380997, 16676365, 16849682, 17079134, 16278815, …	FGFR1: inhibitor; FGFR2: inhibitor; FGFR3: inhibitor; FGFR4: inhibitor; KIT: inhibitor; LYN: inhibitor; PDGFRA: inhibitor; RET: inhibitor; SRC: inhibitor	The most common non-hematologic adverse reactions (≥20%) were hypertension, rash, abdominal pain, fatigue, headache, dry skin, constipation, arthralgia, nausea, and pyrexia. Hematologic adverse reactions included thrombocytopenia, anemia, neutro…
pralsetinib	0.004258	FGFR1, FGFR2, JAK2, NTRK1, NTRK3, RET	PIK3CA	AMPLIFICATION, SOMATIC	118	11370534, 11216646, 12743496, 12670889, 11706744, …	34	17549376, 11358835, 15543611, 16380997, 18376308, …	FGFR1: inhibitor; FGFR2: inhibitor; JAK2: inhibitor; NTRK1: inhibitor; NTRK3: inhibitor; RET: inhibitor	Pralsetinib administered to rats at 20 mg/kg (roughly 2.5–3.6 times the recommended human exposure) resulted in resorption of litters in pregnant female mice in 92% of pregnancies (82% complete resorption); resorption occurred at doses as low as…
pp-121	0.004258	EGFR, HCK, MTOR, PDGFRA, SRC	CDKN2A, PIK3CA, TP53	AMPLIFICATION, DELETION, SOMATIC	915	17949783, 16890793, 18045070, 12599217, 11939730, …	79	16380997, 16676365, 16682429, 16849682, 12702551, …	EGFR: inhibitor; HCK: inhibitor; MTOR: inhibitor; PDGFRA: inhibitor; SRC: inhibitor	None
regorafenib	0.004258	BRAF, EPHA2, FGFR1, FGFR2, KIT, NTRK1, PDGFRA, RET	CLDN1, PIK3CA, TP53	AMPLIFICATION, SOMATIC	217	11370534, 17525478, 17471236, 17638058, 16551863, …	65	16380997, 16676365, 16682429, 12702551, 17638058, …	BRAF: inhibitor; EPHA2: inhibitor; FGFR1: inhibitor; FGFR2: inhibitor; KIT: inhibitor; NTRK1: inhibitor; PDGFRA: inhibitor; RET: inhibitor	The most common adverse reactions (≥20%) are asthenia/fatigue, HFSR, diarrhea, decreased appetite/food intake, hypertension, mucositis, dysphonia, and infection, pain (not otherwise specified), decreased weight, gastrointestinal and abdominal pai…
ponatinib	0.004258	FGFR1, FGFR2, FGFR3, FGFR4, KIT, LYN, PDGFRA, RET, SRC	CDKN2A, PIK3CA	AMPLIFICATION, DELETION, SOMATIC	144	11370534, 16757203, 17935283, 11216646, 12743496, …	50	16380997, 16676365, 16849682, 17079134, 16278815, …	FGFR1: inhibitor; FGFR2: inhibitor; FGFR3: inhibitor; FGFR4: inhibitor; KIT: inhibitor; LYN: inhibitor; PDGFRA: inhibitor; RET: inhibitor; SRC: inhibitor	The most common non-hematologic adverse reactions (≥20%) were hypertension, rash, abdominal pain, fatigue, headache, dry skin, constipation, arthralgia, nausea, and pyrexia. Hematologic adverse reactions included thrombocytopenia, anemia, neutro…
acetylsalicylic acid	0.004258	CCND1, MYC, NFKBIA, PCNA, PTGS2, TP53	HRAS, PIK3CA, TP53	AMPLIFICATION, SOMATIC	114	11368094, 17487429, 17638058, 14499691, 16001430, …	47	16380997, 16682429, 16676365, 12702551, 17638058, …	CCND1: downregulator; MYC: downregulator; NFKBIA: inhibitor; PCNA: downregulator; PTGS2: inhibitor; TP53: inducer	Lethal Doses: Acute oral LD50 values have been reported as over 1.0 g/kg in humans, cats, and dogs, 0.92 g/kg - 1.48 g/kg in albino rats, 1.19 g/kg in guinea pigs, 1.1 g/kg in mice, and 1.8 g/kg in rabbit models [FDA label].
bisindolylmaleimide i	0.004258	AKT1, CDK1, CHEK1, MAPK8, PRKCI	CDKN2A, PIK3CA, RFC4, SOX2, TP53	AMPLIFICATION, DELETION, SOMATIC	35	17974918, 17990328, 12532415, 15896313, 15645429, …	83	16865240, 16380997, 16676365, 16682429, 16849682, …	AKT1: inhibitor; CDK1: inhibitor; CHEK1: inhibitor; MAPK8: inhibitor; PRKCI: UNKNOWN	None
dasatinib	0.004258	EPHB4, FYN, HSPA8, KIT, LYN, SRC, STAT5B, YES1	EPHA2, HRAS, PIK3CA, TP53	AMPLIFICATION, SOMATIC	18	16757203, 17253141, 17317803, 17935283, 17961551, …	52	16380997, 16682429, 16676365, 12702551, 17638058, …	EPHB4: inhibitor; FYN: inhibitor; HSPA8: binder; KIT: antagonist; LYN: inhibitor; SRC: inhibitor; STAT5B: inhibitor; YES1: inhibitor	Overdose cases with dasatinib occurred in isolated cases during clinical studies. Patients that received 280 mg of dasatinib per day for 1 week developed severe myelosuppression and bleeding. Since dasatinib is associated with severe myelosuppres…
ponatinib	0.004258	FGFR1, FGFR2, FGFR3, FGFR4, KIT, LYN, PDGFRA, RET, SRC	CDKN2A, PIK3CA	AMPLIFICATION, DELETION, SOMATIC	144	11370534, 16757203, 17935283, 11216646, 12743496, …	50	16380997, 16676365, 16849682, 17079134, 16278815, …	FGFR1: inhibitor; FGFR2: inhibitor; FGFR3: inhibitor; FGFR4: inhibitor; KIT: inhibitor; LYN: inhibitor; PDGFRA: inhibitor; RET: inhibitor; SRC: inhibitor	The most common non-hematologic adverse reactions (≥20%) were hypertension, rash, abdominal pain, fatigue, headache, dry skin, constipation, arthralgia, nausea, and pyrexia. Hematologic adverse reactions included thrombocytopenia, anemia, neutro…
fostamatinib	0.004258	ALK, AURKA, BRAF, CDK1, CDK4, CHEK1, DAPK1, EGFR, EPHA1, EPHA2, EPHB1, EPHB4, ERBB2, ERBB4, FGFR1, FGFR2, FGFR3, FYN, HCK, JAK2, JAK3, KIT, LYN, MAP2K3, MET, MTOR, NTRK1, NTRK3, PAK1, PDGFRA, PLK1, PLK3, PRKCI, RET, SRC, SYK, WEE1, YES1	CLDN1, EIF4G1, EPHA2, HRAS, PIK3CA, RAC1, RFC4, SOX2, TP53	AMPLIFICATION, SOMATIC	1008	17949783, 16890793, 18045070, 12599217, 16054751, …	80	17234718, 16865240, 16380997, 16676365, 16682429, …	ALK: inhibitor; AURKA: inhibitor; BRAF: inhibitor; CDK1: inhibitor; CDK4: inhibitor; CHEK1: inhibitor; DAPK1: inhibitor; EGFR: inhibitor; EPHA1: inhibitor; EPHA2: inhibitor; EPHB1: inhibitor; EPHB4: inhibitor; ERBB2: inhibitor; ERBB4: inhibitor; …	Neither fostamatinib or R406 were found to be carcinogenic or mutagenic [FDA Label]. Fostamatinib can cause embryo-fetal mortality or developmental abnormalities at exposures of 0.3–10 times the maximum recommended human dose.
ponatinib	0.004258	FGFR1, FGFR2, FGFR3, FGFR4, KIT, LYN, PDGFRA, RET, SRC	CDKN2A, PIK3CA	AMPLIFICATION, DELETION, SOMATIC	144	11370534, 16757203, 17935283, 11216646, 12743496, …	50	16380997, 16676365, 16849682, 17079134, 16278815, …	FGFR1: inhibitor; FGFR2: inhibitor; FGFR3: inhibitor; FGFR4: inhibitor; KIT: inhibitor; LYN: inhibitor; PDGFRA: inhibitor; RET: inhibitor; SRC: inhibitor	The most common non-hematologic adverse reactions (≥20%) were hypertension, rash, abdominal pain, fatigue, headache, dry skin, constipation, arthralgia, nausea, and pyrexia. Hematologic adverse reactions included thrombocytopenia, anemia, neutro…
pazopanib	0.004930	FGFR3, KIT, PDGFRA	PIK3CA	AMPLIFICATION, SOMATIC	24	16757203, 17253141, 17317803, 17935283, 11605053, …	39	16380997, 16676365, 18485799, 16309192, 18030354, …	FGFR3: inhibitor; KIT: inhibitor; PDGFRA: inhibitor	None
quercetin	0.004930	ESR1, HCK, STAT3	PIK3CA	AMPLIFICATION, SOMATIC	44	16158919, 17122156, 11426647, 17145757, 17961551, …	17	17549376, 11358835, 15543611, 16380997, 18376308, …	ESR1: UNKNOWN; HCK: UNKNOWN; STAT3: inhibitor	None
fn-1501	0.006245	CDK2, CDK4, CDK6	CDKN2A, CDKN2B	DELETION, SOMATIC	33	17477349, 12480548, 11267947, 15833870, 15543230, …	18	12684406, 15083191, 17117177, 17079134, 16278815, …	CDK2: inhibitor; CDK4: inhibitor; CDK6: inhibitor	None
olomoucine	0.006245	CDK1, CDK2, CDK5	CDKN2A, CDKN2B	DELETION, SOMATIC	16	16248248, 16496417, 17145757, 12480548, 11267947, …	18	12684406, 15083191, 17117177, 17079134, 16278815, …	CDK1: binder; CDK2: inhibitor; CDK5: inhibitor	None
purvalanol	0.006245	CDK1, CDK2, CDK4, CDK5	CDKN2A, CDKN2B	DELETION, SOMATIC	30	17477349, 17145757, 12480548, 11267947, 15833870, …	18	12684406, 15083191, 17117177, 17079134, 16278815, …	CDK1: binder; CDK2: inhibitor; CDK4: UNKNOWN; CDK5: inhibitor	None
ponatinib	0.004258	FGFR1, FGFR2, FGFR3, FGFR4, KIT, LYN, PDGFRA, RET, SRC	CDKN2A, PIK3CA	AMPLIFICATION, DELETION, SOMATIC	144	11370534, 16757203, 17935283, 11216646, 12743496, …	50	16380997, 16676365, 16849682, 17079134, 16278815, …	FGFR1: inhibitor; FGFR2: inhibitor; FGFR3: inhibitor; FGFR4: inhibitor; KIT: inhibitor; LYN: inhibitor; PDGFRA: inhibitor; RET: inhibitor; SRC: inhibitor	The most common non-hematologic adverse reactions (≥20%) were hypertension, rash, abdominal pain, fatigue, headache, dry skin, constipation, arthralgia, nausea, and pyrexia. Hematologic adverse reactions included thrombocytopenia, anemia, neutro…
tetrabromo-2-benzotriazole	0.006855	AKT1, CHEK1, MAPK8	CDKN2A, PIK3CA, RFC4, TP53	AMPLIFICATION, DELETION, SOMATIC	24	17974918, 12532415, 15896313, 15226608, 16778075, …	82	16865240, 16380997, 16676365, 16682429, 16849682, …	AKT1: inhibitor; CHEK1: inhibitor; MAPK8: inhibitor	None
seliciclib	0.006939	CDK1, CDK2	TP53	SOMATIC	15	16248248, 16496417, 12480548, 11267947, 15833870, …	46	16380997, 16682429, 16676365, 12702551, 17638058, …	CDK1: inhibitor; CDK2: inhibitor	None
ci-1040	0.006939	AKT1, CHEK1, MAPK8	CDKN2A, PIK3CA, RFC4, TP53	AMPLIFICATION, DELETION, SOMATIC	24	17974918, 12532415, 15896313, 15226608, 16778075, …	82	16865240, 16380997, 16676365, 16682429, 16849682, …	AKT1: inhibitor; CHEK1: inhibitor; MAPK8: inhibitor	None
resveratrol	0.006939	AKT1, APP, ESR1, ITGB3, PTGS2	COL1A2, PIK3CA, TP53	AMPLIFICATION, SOMATIC	10	17974918, 12532415, 17679464, 18030354, 15896313, …	47	16380997, 16682429, 16676365, 12702551, 17638058, …	AKT1: inhibitor; APP: UNKNOWN; ESR1: UNKNOWN; ITGB3: UNKNOWN; PTGS2: inhibitor	None
arsenic trioxide	0.006939	AKT1, CCND1, CDKN1A, HDAC1, PML	CDKN2A, CDKN2B, PIK3CA, TP53	AMPLIFICATION, DELETION, SOMATIC	67	17974918, 15896313, 16490596, 15195525, 15846092, …	64	16380997, 16682429, 16676365, 17673925, 16849682, …	AKT1: inducer; CCND1: antagonist; CDKN1A: UNKNOWN; HDAC1: UNKNOWN; PML: UNKNOWN	Symptoms of overdose include convulsions, muscle weakness and confusion.
midostaurin	0.008516	KIT, PDGFRA	PIK3CA	AMPLIFICATION, SOMATIC	18	17253141, 16757203, 17317803, 17935283, 16630292, …	34	17549376, 11358835, 15543611, 16380997, 18376308, …	KIT: antagonist; PDGFRA: antagonist	In a fertility study involving female and male rats, there is evidence of reproductive toxicity including reduced sperm count and decline pregnancy rates when administering 0.01 to 0.1 times the recommended dose in humans. Incidences of pulmonary…
semaxanib	0.008516	FGFR1, KIT, PDGFRA, RET	PIK3CA	AMPLIFICATION, SOMATIC	61	11370534, 16757203, 17935283, 11216646, 12743496, …	17	17549376, 11358835, 15543611, 16380997, 18376308, …	FGFR1: inhibitor; KIT: inhibitor; PDGFRA: inhibitor; RET: inhibitor	None
hymenialdisine	0.010409	CDK1, CDK2, CDK5	CDKN2A, CDKN2B	DELETION, SOMATIC	16	16248248, 16496417, 17145757, 12480548, 11267947, …	18	12684406, 15083191, 17117177, 17079134, 16278815, …	CDK1: UNKNOWN; CDK2: inhibitor; CDK5: inhibitor	None
su9516	0.010409	CDK1, CDK2, CDK5	CDKN2A, CDKN2B	DELETION, SOMATIC	16	16248248, 16496417, 17145757, 12480548, 11267947, …	18	12684406, 15083191, 17117177, 17079134, 16278815, …	CDK1: binder; CDK2: inhibitor; CDK5: inhibitor	None
martinostat	0.013952	HDAC1	BCL6	AMPLIFICATION	1	12239610	4	11224600, 14685876, 11420458, 17429099	HDAC1: inhibitor	None
tivozanib	0.016152	FGFR1, KIT, MET, PDGFRA	PIK3CA	AMPLIFICATION, SOMATIC	50	16757203, 17075124, 17935283, 17513510, 17684930, …	34	17549376, 11358835, 15543611, 16380997, 18376308, …	FGFR1: inhibitor; KIT: inhibitor; MET: inhibitor; PDGFRA: inhibitor	LD50 information for tivozanib is not readily available in the literature, however the European Medicines Agency (EMA) assessment report indicates the oral maximum tolerated dose (MTD) in mice was 524 mg/kg; doses ≥750 mg/kg led to severe and le…
ripretinib	0.016532	BRAF, KIT, PDGFRA	PIK3CA	AMPLIFICATION, SOMATIC	49	16757203, 15613458, 16728576, 17525478, 17935283, …	17	17549376, 11358835, 15543611, 16380997, 18376308, …	BRAF: inhibitor; KIT: inhibitor; PDGFRA: inhibitor	There is limited information in the literature regarding an overdose with ripretinib, and LD50 information is not readily available. [L13769] Like other kinase inhibitors, an overdose with ripretinib likely results in hematological toxicity, skin…
famitinib	0.018131	KIT, PDGFRA	PIK3CA	AMPLIFICATION, SOMATIC	18	17253141, 16757203, 17317803, 17935283, 16630292, …	34	17549376, 11358835, 15543611, 16380997, 18376308, …	KIT: inhibitor; PDGFRA: inhibitor	None
artenimol	0.018131	ANXA2, GAPDH, HSPA8, LGALS1, PRDX1, RPS13, RPS6, VIM	EIF4G1, HRAS, SOX2, TP53	AMPLIFICATION, SOMATIC	24	16761435, 17884789, 16278420, 17786348, 17132224, …	85	17234718, 16380997, 16676365, 16682429, 16849682, …	ANXA2: ligand; GAPDH: ligand; GAPDH: ligand; HSPA8: ligand; LGALS1: ligand; PRDX1: ligand; RPS13: ligand; RPS6: ligand; VIM: ligand	In single dose studies in CD-1 male mice, Artenimol did not show signs of toxicity up to 1200 mg/kg [L1149]. In repeated dose studies in CD-1 mice, the NOAEL for Artenimol was determined to be 200 mg/kg/day [L1149]. This value was determin…
ag-24322	0.018736	CDK1, CDK2, CDK4	CDKN2A, CDKN2B	DELETION, SOMATIC	29	17477349, 12480548, 11267947, 15833870, 15645429, …	18	12684406, 15083191, 17117177, 17079134, 16278815, …	CDK1: inhibitor; CDK2: inhibitor; CDK4: inhibitor	None
mkc-1	0.018736	CDK1, CDK2, CDK4	CDKN2A, CDKN2B	DELETION, SOMATIC	29	17477349, 12480548, 11267947, 15833870, 15645429, …	18	12684406, 15083191, 17117177, 17079134, 16278815, …	CDK1: inhibitor; CDK2: inhibitor; CDK4: inhibitor	None
pd-168393	0.018736	EGFR, ERBB2, SRC	CDKN2A	DELETION, SOMATIC	321	17949783, 16890793, 18045070, 12599217, 11939730, …	16	12684406, 15083191, 17117177, 17079134, 16278815, …	EGFR: inhibitor; ERBB2: inhibitor; SRC: UNKNOWN	None
pha-793887	0.018736	CDK1, CDK2, CDK4	CDKN2A, CDKN2B	DELETION, SOMATIC	29	17477349, 12480548, 11267947, 15833870, 15645429, …	18	12684406, 15083191, 17117177, 17079134, 16278815, …	CDK1: inhibitor; CDK2: inhibitor; CDK4: inhibitor	None
enzastaurin	0.020074	AKT1, AURKA, CHEK1	CDKN2A, PIK3CA, TP53	AMPLIFICATION, DELETION, SOMATIC	22	17974918, 12532415, 15896313, 18311783, 16778075, …	79	16380997, 16676365, 16682429, 16849682, 12702551, …	AKT1: inhibitor; AURKA: UNKNOWN; CHEK1: UNKNOWN	None
prasterone	0.024089	AR, ESR1	PIK3CA, TP53	AMPLIFICATION, SOMATIC	11	16690481, 18528886, 17453000, 17017191, 12817426, …	46	16380997, 16682429, 16676365, 12702551, 17638058, …	AR: agonist; ESR1: binder	Acute oral toxicity (LD50): >10,000 mg/kg [Rat]. Lowest Published Toxic Dose (TDL) [Man]-Route: Oral; Dose: 10 mg/kg/2 W intermittent.
trilaciclib	0.024089	CDK2, CDK4, CDK5, CDK6	CDKN2A, CDKN2B, PIK3CA, TP53	AMPLIFICATION, DELETION, SOMATIC	68	17477349, 17145757, 12480548, 11267947, 15833870, …	64	16380997, 16682429, 16676365, 17673925, 16849682, …	CDK2: inhibitor; CDK4: inhibitor; CDK5: inhibitor; CDK6: inhibitor	Data regarding overdoses of trilaciclib are not readily available. [L31828] For grade 3 or worse injection site reactions, acute drug hypersensitivity reactions, and interstitial lung disease; or any other grade 4 toxicities; permanently discontin…
selpercatinib	0.028624	FGFR1, FGFR2, FGFR3, RET	PIK3CA	AMPLIFICATION, SOMATIC	114	11370534, 11216646, 12743496, 12670889, 11706744, …	34	17549376, 11358835, 15543611, 16380997, 18376308, …	FGFR1: inhibitor; FGFR2: inhibitor; FGFR3: inhibitor; RET: inhibitor	Toxicity information regarding selpercatinib is not readily available. Patients experiencing an overdose are at an increased risk of severe adverse effects such as hepatotoxicity, hypertension, prolonged QT interval, and hemorrhaging. Symptomatic…
amuvatinib	0.028824	KIT, MET, PDGFRA, RAD51, RET	PIK3CA, TP53	AMPLIFICATION, SOMATIC	136	11370534, 16757203, 17075124, 17935283, 11216646, …	63	16380997, 16676365, 16682429, 12702551, 17638058, …	KIT: modulator; MET: UNKNOWN; PDGFRA: modulator; RAD51: UNKNOWN; RET: UNKNOWN	None
linifanib	0.028824	KIT	PIK3CA	AMPLIFICATION, SOMATIC	14	17253141, 16757203, 17317803, 17935283, 16630292, …	34	17549376, 11358835, 15543611, 16380997, 18376308, …	KIT: UNKNOWN	None
xl999	0.028824	FGFR1, FGFR3, RET	PIK3CA	AMPLIFICATION, SOMATIC	55	11370534, 11216646, 12743496, 12670889, 11706744, …	17	17549376, 11358835, 15543611, 16380997, 18376308, …	FGFR1: UNKNOWN; FGFR3: UNKNOWN; RET: UNKNOWN	None
canertinib	0.028824	AKT1, EGFR, ERBB2	CDKN2A	DELETION, SOMATIC	326	17949783, 16890793, 18045070, 12599217, 11939730, …	16	12684406, 15083191, 17117177, 17079134, 16278815, …	AKT1: inhibitor; EGFR: inhibitor; ERBB2: inhibitor	None
alsterpaullone	0.030111	CDK1, CDK2, CDK5	CDKN2A, CDKN2B	DELETION, SOMATIC	16	16248248, 16496417, 17145757, 12480548, 11267947, …	18	12684406, 15083191, 17117177, 17079134, 16278815, …	CDK1: inhibitor; CDK2: inhibitor; CDK5: inhibitor	None
zanubrutinib	0.030714	EGFR, ERBB2, ERBB4, JAK2, JAK3	PIK3CA	AMPLIFICATION, SOMATIC	327	17949783, 16890793, 18045070, 12599217, 11939730, …	17	17549376, 11358835, 15543611, 16380997, 18376308, …	EGFR: inhibitor; ERBB2: inhibitor; ERBB4: inhibitor; JAK2: inhibitor; JAK3: inhibitor	There is limited data on zanubrutinib overdose.
foreskin keratinocyte (neonatal)	0.037918	EGFR, FGFR2, IL1B, IL6, PDGFRA, TNF	EPHA2, PIK3CA, TP53	AMPLIFICATION, SOMATIC	308	17949783, 18197435, 16890793, 18045070, 12599217, …	51	16380997, 16682429, 16676365, 12702551, 17638058, …	EGFR: agonist; FGFR2: agonist; IL1B: agonist; IL6: agonist; PDGFRA: agonist; TNF: agonist	Adverse events in a preclinical study of about 300 subjects were classified as mild, moderate, severe or life-threatening. In the venous leg ulcer study, 1 life-threatening infection and 3 severe infections reported in the Apligraf group occurred…
